# Immediate Circulating Cortisol Responses Differ Between Two Functionally Divergent Localized Resistance-Type Plantar Flexion and Elbow Flexions: An Exploratory Within-Participant Crossover Study

**DOI:** 10.3390/life16091525

**Published:** 2026-09-14

**Authors:** Anna Adam, Łukasz Słomski, Jolanta Smykiewicz, Iwona Bachman, Igor Z. Zubrzycki, Magdalena Wiacek

**Affiliations:** 1 Faculty of Medical and Health Sciences, Casimir Pulaski Radom University, ul. Chrobrego 27, 26-660 Radom, Poland; 2 Central Medical Diagnostic Laboratory, Dr. Tytus Chałubiński Specialist Hospital in Radom, ul. Lekarska 4, 26-610 Radom, Poland; 3 Department of Health Sciences, Kujawska University in Włocławek, ul. Okrzei 94A, 87-800 Włocławek, Poland

**Keywords:** skeletal muscle metabolism, localized resistance exercise, systemic biomarkers, glucose response, cortisol, exercise biochemistry

## Abstract

Background: Practical localized resistance tasks involving functionally divergent muscle groups may produce different systemic biochemical responses, although neither task isolates a single muscle. Methods: In this exploratory, nonrandomized, order-balanced within-participant crossover study, 102 young adults completed bilateral seated plantar flexion and unilateral elbow flexion, with 51 participants assigned by alternation to each condition sequence. The analysis-ready biochemical dataset contained complete observations for 102 participants. Blood was collected before and 2–3 min after each condition. We analyzed plasma glucose and lactate and serum total cholesterol, HDL-C, LDL-C, triglycerides, creatine kinase, lactate dehydrogenase, GGT, and cortisol. Linear mixed-effects models included exercise condition, sampling time, study period, assigned sequence, and their respective interactions with sampling time as fixed effects. Participant and participant-specific study visit were included as random intercepts where supported by the model. The exercise condition × time interaction remained the primary contrast, and Holm correction was applied across the ten predefined biochemical outcomes. Linear mixed-effects models included exercise condition, sampling time, study period, assigned sequence, and their respective interactions with sampling time as fixed effects. Participant and participant-specific study visit were included as random intercepts where supported by the model. The exercise condition × time interaction remained the primary contrast, and Holm correction was applied across the ten predefined biochemical outcomes. Results: After adjustment for period and sequence and Holm correction across the ten primary interaction tests, cortisol remained the only statistically significant exercise condition × time interaction (149.219 nmol·L^−1^, 95% CI 62.338 to 236.099; unadjusted *p* = 0.000821; Holm-adjusted *p* = 0.008). Glucose showed a negative interaction estimate (−7.013 mg·dL^−1^, 95% CI −12.238 to −1.788; unadjusted *p* = 0.009), but did not remain significant after Holm correction (adjusted *p* = 0.078). No period × time or sequence × time effect remained statistically significant after correction. Sensitivity analyses were not fully concordant with the primary model, supporting cautious interpretation of the cortisol finding. Conclusions: These findings describe immediate responses to two non-equivalent multi-muscle tasks and cannot be attributed to selective soleus or biceps-brachii activation, fiber-type composition, or muscle-specific substrate use.

## 1. Introduction

Skeletal muscle is central to whole-body metabolic regulation [1,2], but its contribution to systemic biochemical responses is not uniform across muscles. Individual muscles differ in habitual function, recruitment pattern, fiber-type distribution, oxidative capacity, vascularization, and substrate preference [3]. These local differences are well established at the histological, molecular, and enzymatic levels, yet it remains uncertain whether they are detectable through routine circulating biomarkers after practical localized plantar flexion and elbow flexion.

This question is relevant because circulating markers such as glucose, lactate, lipids, enzymes, and cortisol are accessible, clinically familiar, and feasible for applied exercise studies [4,5]. However, these markers reflect integrated whole-body regulation rather than isolated intramuscular metabolism. Therefore, determining whether localized plantar flexion and elbow flexion involving functionally divergent muscle groups produce distinguishable systemic biochemical responses may help clarify both the potential and the limits of blood-based monitoring after localized exercise [5].

Skeletal muscles are not metabolically homogeneous; instead, they exhibit pronounced heterogeneity in fiber-type composition, enzymatic activity, and substrate utilization, which together shape their aerobic and anaerobic metabolic profiles [6,7].

In humans, the soleus is characterized by a relatively high but variable proportion of type I fibers, commonly reported within approximately 45–65%, together with high oxidative enzyme activity and a predominant role in sustained postural activity [8]. Functionally, the soleus plays a key role in postural control and sustained low-intensity contractions and is well suited for oxidative metabolism, including the use of circulating glucose and fatty acids. However, substrate selection depends on exercise intensity, duration, and nutritional state [9,10,11].

In contrast, the biceps brachii is a non-postural, phasic upper-limb muscle with mixed fiber-type composition and a greater contribution of type II fibers than the soleus [12,13,14]. This organization supports rapid voluntary force production and repeated phasic movements, with a relatively greater reliance on glycolytic energy pathways during sufficiently intense contractions [12]. During high-intensity contractions, greater recruitment of type II fibers is generally associated with a larger contribution from glycolytic ATP provision; however, actual substrate use depends strongly on relative intensity, contraction duration, and recruitment pattern [13].

Although extensive literature describes differences in fiber-type composition, oxidative enzyme activity, mitochondrial density, and substrate utilization among skeletal muscles, it remains uncertain whether these local characteristics translate into measurable systemic biochemical responses after practical localized resistance-type exercise. Most previous studies have relied on histological classification, gene-expression profiling, intramuscular enzymatic assays, or whole-body exercise models rather than direct comparisons of localized plantar flexion and elbow flexions using circulating biochemical endpoints [6,7,15]. Whole-body exercise studies have established that skeletal muscle is a major determinant of lipid and glucose turnover [16], and recent work highlights the potential metabolic relevance of oxidative postural muscles such as the soleus in systemic glucose and lipid regulation [17]. However, direct experimental comparisons of localized plantar flexion and elbow flexions emphasizing functionally divergent muscle groups within the same physiological framework remain limited. Thus, a paired comparison of localized plantar flexion and elbow flexions may help determine whether routine circulating markers are sufficiently sensitive to detect immediate systemic differences between functionally divergent exercise conditions.

A central conceptual issue is whether circulating biochemical markers can detect localized muscle-related metabolic differences. Plasma glucose, lipids, lactate, enzymes, and cortisol do not directly quantify intramuscular substrate oxidation or fiber-type-specific metabolism. Rather, they reflect integrated whole-body regulation, including skeletal muscle uptake, hepatic output, adipose tissue flux, endocrine regulation, plasma volume shifts, clearance kinetics, and inter-individual variability. Therefore, the present study was not designed to isolate fiber-type composition as an independent determinant of metabolic response. Instead, these physiological differences served as the biological rationale for selecting two practical localized resistance-type tasks, while the experimental question was whether the two complete exercise conditions produced distinguishable systemic biochemical response patterns [18,19].

The present study compared two practical localized resistance-type exercise conditions: bilateral seated plantar flexion performed with the knee flexed and unilateral seated elbow flexion. These tasks were selected because they emphasize functionally divergent muscle groups. Nevertheless, neither task isolates an individual muscle. Seated plantar flexion involves the soleus together with contributions from the gastrocnemius and other plantar flexors, whereas elbow flexion involves the biceps brachii, brachialis, brachioradialis, stabilizing muscles, and potential antagonist coactivation. No direct measurement of relative muscle recruitment was performed.

Accordingly, the aim was to determine whether the immediate systemic biochemical response differed between two standardized but mechanically and physiologically non-equivalent localized resistance-type exercises: plantar flexion and elbow flexion. The primary statistical contrast was the exercise condition × time interaction across the predefined biochemical panel. The study was not designed to quantify muscle activation, isolate individual-muscle metabolism, or test fiber-type-specific substrate utilization. Therefore, the findings were interpreted as condition-dependent systemic responses to the applied plantar flexion and elbow flexions rather than as direct evidence of soleus- or biceps-brachii-specific metabolism.

## 2. Materials and Methods

### 2.1. Study Design

The study employed a nonrandomized, order-balanced within-participant crossover design in which every participant completed both exercise conditions. Consecutively enrolled participants were assigned by alternation to the plantar-flexion–elbow-flexion sequence or the elbow-flexion–plantar-flexion sequence, with 51 participants assigned to each sequence. Because alternation was predictable and allocation was not concealed, the design should not be interpreted as randomized. Sessions were separated by an inter-session interval of 48–72 h. For the crossover analysis, the first and second experimental sessions were coded as periods 1 and 2, respectively. Assigned order was coded as the plantar-flexion → elbow-flexion sequence or the elbow-flexion → plantar-flexion sequence. We reconstructed period and sequence from the documented alternating allocation order and participant enrollment sequence, yielding 51 participants in each sequence. Because every participant completed both conditions, the design enabled paired within-participant comparison of exercise-induced biochemical changes.

The study was exploratory and based on systemic profiling. No individual circulating biomarker was designated as a standalone primary endpoint. Instead, the biochemical variables were selected to represent complementary domains of systemic response, including lipid metabolism, glucose regulation, lactate response, enzymatic activity, hepatic-associated enzyme activity, and endocrine response. The primary statistical contrast was the exercise condition × time interaction across the predefined biochemical panel.

Because multiple biochemical endpoints were analyzed, we addressed the risk of type I error inflation by defining the exercise condition × time interaction as the primary inferential test for each outcome and applying Holm correction across the predefined family of primary interaction tests. We interpreted statistically significant findings only after multiplicity correction, while describing unadjusted trends cautiously as exploratory.

Plantar flexion and elbow flexions were standardized for external load, anatomical position, task termination criteria, and verbal encouragement, but were not normalized to individual maximal voluntary strength, relative intensity, total mechanical work, or electromyographic activation. The protocol was therefore designed to provide standardized practical localized exercise exposures rather than to impose identical mechanical workload, equivalent relative intensity, or uniform exhaustion across muscles.

### 2.2. Participants

We screened 112 individuals aged 18–29 years from the local university community using convenience sampling. Ten individuals who presented with active menstrual bleeding on a scheduled testing day were not enrolled in the experimental sessions, leaving 102 participants who completed both conditions. This criterion was applied in the original protocol; however, menstrual-cycle phase and reproductive-hormone concentrations were not assessed in the included participants. No specific physiological rationale for this exclusion criterion was prespecified in the study documentation; it is therefore reported transparently as a protocol-specific restriction rather than as a method of controlling menstrual-cycle physiology.

Consequently, the exclusion did not control for menstrual-cycle-related physiological variation and may have introduced sex-specific selection bias. The inclusion criteria required the absence of diagnosed cardiovascular, metabolic, endocrine, or neuromuscular disease.

Additional exclusion criteria included: (1) current use of medications known to influence lipid metabolism, glucose regulation, or endocrine function, including lipid-lowering agents, glucose-lowering medications, corticosteroids, or hormonal treatments; (2) current smoking; (3) musculoskeletal injury or surgery during the preceding six months; and (4) any condition limiting safe participation in resistance-type exercise. Menstrual-cycle phase and circulating reproductive-hormone concentrations were not measured, and residual menstrual-cycle-related physiological variation cannot be excluded.

We recorded sex, age, BMI, waist-to-height ratio, and habitual physical-activity category for descriptive characterization of the study cohort. Because the study used a within-participant crossover design, stable participant characteristics were held constant across exercise conditions. These variables were not included in the final inferential models.

To reduce between-session variation in short-term nutrient intake and metabolic substrate availability, participants received a standardized seven-day meal list. They were instructed to follow it before the first experimental session and throughout the interval between sessions. The standardized menu for the day immediately preceding measurement was repeated before both experimental sessions. The complete meal list and accompanying dietary instructions are presented in Supplementary Table S1. Food portions were communicated using household measures; meals were not weighed, and total energy and macronutrient intakes were not quantitatively calculated. Adherence to the meal list was not independently verified. Therefore, the meal list was treated as a standardized pre-experimental instruction rather than as a quantitatively analyzed dietary intervention.

Participants also completed an 8–12 h overnight fast and avoided strenuous activity, alcohol, and caffeine for 24–48 h, and at least 12 h before each session. Sleep duration, hydration status, and psychological stress were not quantitatively assessed.

All individuals provided written informed consent prior to participation, and the local institutional ethics committee approved the study protocol in accordance with the Declaration of Helsinki (KB/05/2023).

### 2.3. Selection and Interpretation of Exercise Conditions

The term “emphasizing” is used because no exercise selectively activates a single muscle. Seated plantar flexion recruits the soleus together with the gastrocnemius, other plantar flexors, and ankle stabilizers, whereas elbow flexion recruits the biceps brachii together with the brachialis, brachioradialis, and stabilizing muscles. Muscle activation was not measured because the study was designed to compare systemic biochemical responses to the applied plantar flexion and elbow flexions rather than muscle-specific recruitment. Consequently, the condition labels identify the intended exercise emphasis and do not imply isolated activation of a particular muscle.

Two localized resistance-type exercises, plantar flexion and elbow flexion, emphasizing anatomically and functionally divergent muscle groups, were selected to compare systemic biochemical responses. The soleus was selected as a predominantly postural lower-limb muscle with relatively high oxidative capacity and frequent recruitment during standing and locomotor tasks. The biceps brachii was selected as a non-postural upper-limb muscle involved primarily in voluntary phasic movements and force production during elbow flexion. These tasks were therefore used as functionally divergent localized exercise conditions rather than as experimental models of isolated muscle or fiber-type biology.

The seated position for calf raises, with the hips and knees flexed to approximately 90°, was selected to reduce the relative contribution of the gastrocnemius. Because the gastrocnemius is biarticular, knee flexion mechanically shortens the muscle and reduces its capacity to generate plantar-flexion torque. In contrast, the monoarticular soleus remains mechanically advantaged in this position, resulting in preferential activation during seated plantar-flexion tasks [20].

## 3. Experimental Protocol

Exercise-performance variables recorded for each condition included total repetitions, exercise duration, protocol-termination mode and, among participants who met the predefined failure criterion, time to objective task failure. Participants self-selected movement cadence. Participants were instructed to maintain a smooth, consistent cadence throughout the plantar flexion and elbow flexion; however, movement tempo was neither externally paced nor instrumentally quantified.

Ratings of perceived exertion were obtained immediately after each exercise condition using the Borg CR-10 scale. RPE data were available for all 102 participants and were analyzed descriptively.

For both exercise conditions, a 5 min cap was predefined to avoid excessively prolonged low-load activity and to preserve the resistance-type character of the tasks. Protocol termination could occur due to objective task failure or reaching the predefined 5 min cap. Reaching the cap was classified as protocol termination, not objective task failure. Consequently, participants who reached the cap were considered to have completed the standardized exercise exposure without verified objective exhaustion.

Because intensity was not normalized to %MVC, %1RM, power output, total mechanical work, or electromyographic activation level, the protocol should be interpreted as a comparison of two standardized, localized resistance-type exercise exposures, not as a comparison of mechanically, metabolically, or uniformly equivalent task-failure conditions.

Volitional exhaustion was operationally defined as task failure, i.e., the point at which the participant could no longer complete a full repetition through the prescribed range of motion despite standardized verbal encouragement. Task failure was recorded when either (i) a full repetition could not be completed in the required range, or (ii) compensatory movements (e.g., trunk flexion, hip extension, shoulder/trunk swing, incomplete heel elevation) occurred on two consecutive attempts, indicating loss of task-specific motor control. Upon reaching the task failure point, the exercise was immediately terminated. Participants who completed the full 5 min period without meeting the objective task-failure criteria were classified as time-cap completers. This classification was retained for descriptive reporting and interpretive purposes because time-cap completion indicates that the participant did not demonstrate objective biomechanical task failure within the predefined protocol duration.

Verbal encouragement was standardized across sessions and delivered by the same trained investigator using a predefined script and consistent tone. Standardized verbal encouragement was intended to promote sustained effort; however, it could not ensure maximal voluntary effort or equivalent proximity to exhaustion, particularly among participants who reached the time cap without objective task failure.

Workloads were fixed a priori by sex rather than prescribed as a percentage of 1RM or MVC: 10 kg for women and 20 kg for men during the plantar-flexion task, and 2 kg for women and 4 kg for men during the elbow-flexion task. Repetitions were performed at a self-selected cadence until objective task failure or the predefined 5 min cap; consequently, relative intensity and time under tension were not standardized. This approach standardized the external task structure but did not ensure comparable relative intensity, fatigue level, mechanical work, or power output across participants.

Because the exercise conditions differed substantially in external load, activated muscle mass, bilateral versus unilateral performance, joint mechanics, and range of motion, external load × repetitions was treated only as a descriptive load–repetition product. It was not interpreted as total mechanical work or as evidence of equivalent physiological stimulus. Target-muscle activation, antagonist coactivation, and neuromuscular fatigue were not measured.

**Figure 1:**
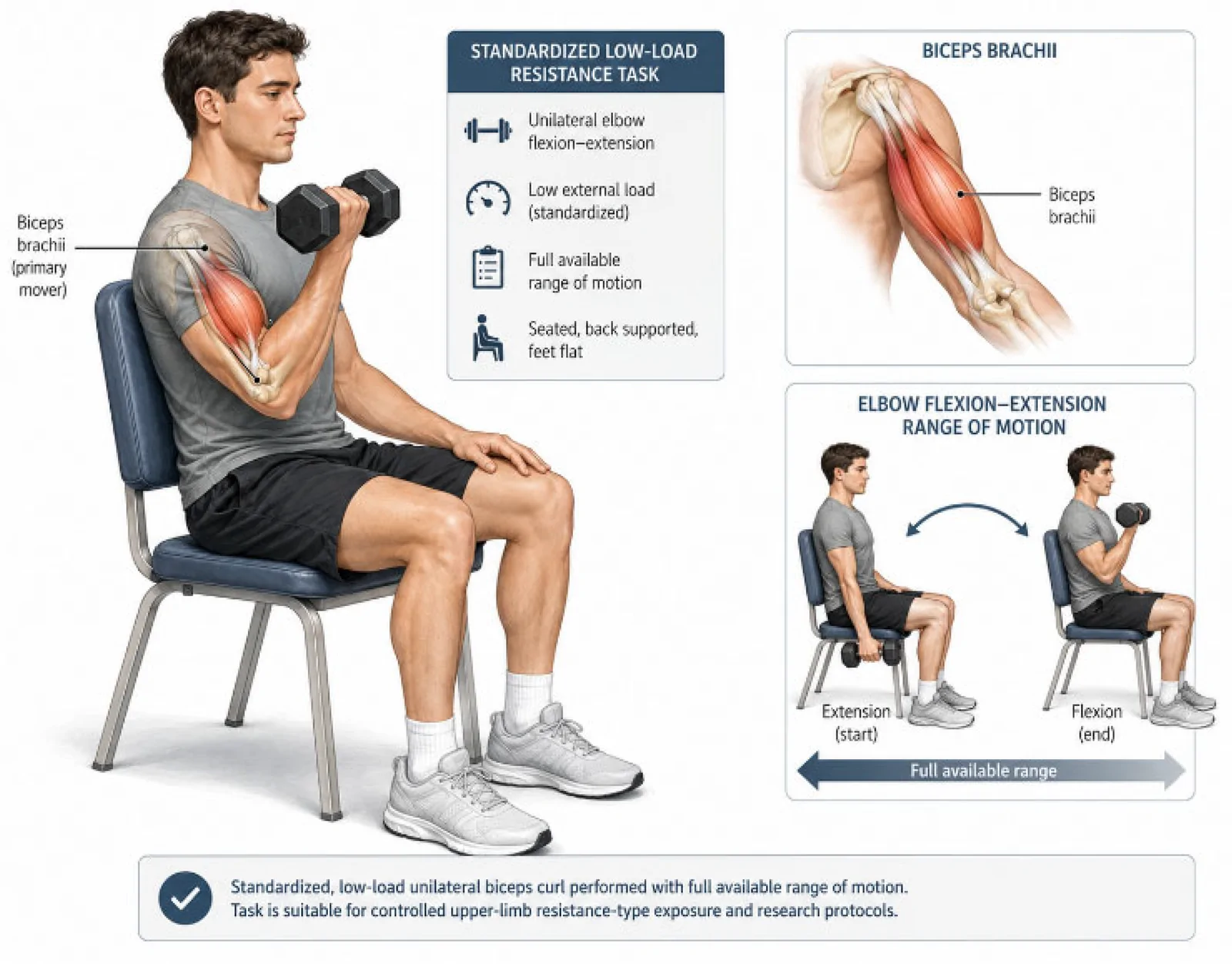
The seated elbow flexion task was performed as a standardized localized resistance-type exposure targeting the biceps brachii. The participant is seated upright in a chair, with the upper arm supported against the thigh and the elbow flexed, while lifting a dumbbell through a controlled range of motion. The primary panel illustrates the exercise posture and external load application, while the inset panels depict the biceps brachii anatomy and the muscle during active contraction. The task was performed repetitively until objective task failure or until the predefined 5 min cap was reached. The illustration presents the intended experimental position and does not represent direct measurement of individual-muscle recruitment.

**Biceps curls:** participants were seated upright on a chair with the trunk supported and both feet flat on the floor. Participants used their dominant upper limb for the task. Participants held a standardized dumbbell (2 kg for women and 4 kg for men) in the dominant hand and were instructed to perform unilateral elbow flexion–extension through the full available range of motion. These conservative standardized loads were selected to permit safe completion of the repeated localized upper-limb task across participants with heterogeneous habitual physical-activity levels. However, the loads were not individualized according to %1RM, %MVC, or maximal elbow-flexor strength. Therefore, the biceps-brachii-emphasizing condition should be interpreted as a standardized low-load upper-limb resistance-type exposure rather than an individualized high-intensity fatiguing protocol (Figure 1).

**Figure 2:**
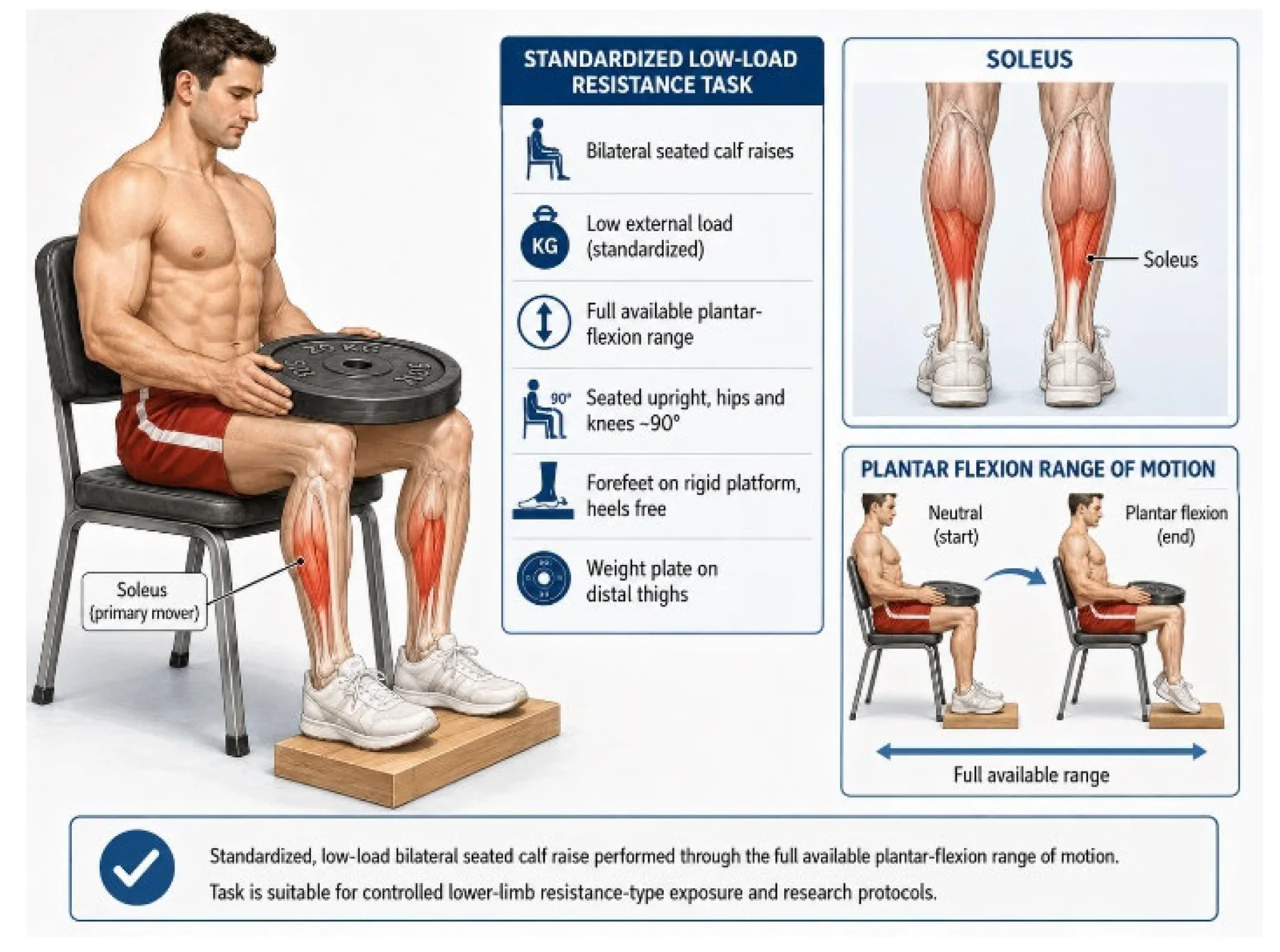
Experimental setup for the soleus-emphasizing bilateral seated plantar-flexion task. The participant is seated upright on a rigid chair with the hips and knees flexed to approximately 90°. The forefeet are placed on a rigid wooden platform positioned beneath the metatarsal heads, allowing unrestricted ankle plantar flexion and full heel excursion while maintaining constant foot placement. External resistance is applied via an Olympic weight plate secured across the distal thighs, proximal to the knees. The task was performed until objective task failure or until the predefined 5 min cap was reached. The illustration presents the intended experimental position and does not represent direct measurement of individual-muscle recruitment.

**Soleus-emphasizing seated calf raises:** participants were seated upright on a rigid chair, with the hips and knees flexed to approximately 90°, to minimize gastrocnemius contribution. Participants placed their forefeet on a rigid wooden platform positioned beneath the metatarsal heads, allowing unrestricted ankle plantar flexion and full heel excursion while maintaining constant foot placement throughout the task. External resistance was applied by placing an Olympic weight plate of predetermined mass (10 kg for women and 20 kg for men) across the distal thighs, immediately proximal to the knees. The load was secured to prevent displacement during movement. Participants were instructed to keep the trunk upright, with hands resting on the thighs, and to avoid compensatory hip or trunk movements (Figure 2). Participants performed bilateral seated calf raises through the full available range of motion, defined as maximal voluntary plantar flexion followed by controlled lowering to the neutral ankle position.

### 3.1. Biochemical Parameters

Participants were instructed to avoid strenuous physical activity and alcohol for 24–48 h before sampling, and to report to the laboratory after an overnight fast of 8–12 h. Participants were not allowed caffeinated beverages for at least 12 h before testing.

To minimize circadian variability, all sessions were performed in the morning (between 09:00 and 11:00). Baseline venous blood was drawn immediately before the exercise bout, after the participant had been seated quietly for a standardized rest period.

The 2–3 min post-exercise sampling window was selected to characterize very early circulating biochemical changes. This interval may capture part of the early glucose response but may precede the peak blood-lactate response and is insufficient to characterize the temporal response of cortisol, CK, or LDH. Accordingly, these measurements represent concentrations observed at a single early recovery time point rather than complete exercise-response kinetics.

Thus, the protocol was not designed to assess delayed muscle damage, delayed enzymatic release, structural remodeling, or the full endocrine recovery profile, which would require serial blood sampling over a longer post-exercise period. Moreover, controlling the post-exercise interval is particularly important for metabolites with rapid kinetics, such as lactate and glucose, which can change substantially within minutes following cessation of muscular activity. Restricting collection to the 2–3 min post-exercise window reduced, but did not eliminate, timing-related variation. Plasma glucose, lactate, and serum TC, HDL-C, LDL-C, TG, CK, LDH, GGT, and cortisol were measured.

#### 3.1.1. Blood Collection and Biochemical Analyses

Qualified medical personnel collected venous blood in the nursing room in accordance with aseptic and biological safety principles. The blood was collected from the cubital vein using disposable aspiration-vacuum S-Monovette sample syringes from Sarstedt. Qualified personnel performed each collection under aseptic conditions.

Blood for glucose and lactate concentration determination was collected from the cubital vein into a 2.7 mL S-Monovette tube (Sarstedt) containing a glycolysis inhibitor (fluoride + EDTA, FE/2.7 mL) to obtain plasma (ref. no. 051073001). The test material (plasma) was obtained by centrifuging the collected blood at 1500× *g* for 10 min using an MPW M-UNIVERSAL laboratory centrifuge (MPW MED. INSTRUMENTS Cooperative, Warsaw, Poland). The plasma was stored at 2–8 °C and transported to a diagnostic laboratory for further preparation and analysis in accordance with laboratory procedures.

For the analysis of TC, HDL-C, LDL-C, TG, LDH, CK, GGT, and CORT, blood was collected into 2.7 mL S-Monovette tubes. These samples were not centrifuged at the collection site. After completion of the collection procedure, all biological material was immediately transported to the diagnostic laboratory, where centrifugation and subsequent sample preparation for analysis were performed in accordance with applicable standards.

All biochemical tests were performed in a certified laboratory using a Roche cobas^®^ pro automatic analyzer (F. Hoffmann-La Roche Ltd, Branchburg, NJ, USA) (biochemical and immunochemical modules) and original reagent kits. The following analytical methods were employed: glucose, hexokinase enzymatic method; lactate, enzymatic method; LDH, CK, and GGT, UV kinetic methods in accordance with IFCC recommendations; TC, HDL-C, LDL-C, and TG, colorimetric enzymatic methods; and cortisol, electrochemiluminescence immunoassay.

#### 3.1.2. Quality Control and Reporting

Internal quality controls at low and high concentration ranges were run at the beginning of each analytical series, according to the manufacturer’s specifications. Glucose, lactate, and lipid concentrations were reported in mg·dL^−1^, enzyme activities in U·L^−1^, and cortisol concentrations in nmol·L^−1^. We used the same units consistently in the statistical analyses, tables, and figures.

### 3.2. Statistical Analysis

All statistical analyses were performed using R version 4.5.0 (R Foundation for Statistical Computing, Vienna, Austria). We fitted linear mixed-effects models using lmerTest (version 4.5.0). We estimated marginal means and condition-specific contrasts using emmeans with Satterthwaite degrees of freedom. Standardized effect sizes and their confidence intervals were calculated using effectsize. Model diagnostics were conducted using performance, and principal component analysis was conducted using FactoMineR 2.16.

Data were imported and processed using openxlsx, readr, dplyr, tidyr, and tibble. Figures were generated using ggplot2, ggdist, and ggbeeswarm, and assembled using magick and grid 2.9.1.

For each biochemical outcome, we analyzed all four observations contributed by each participant jointly. Exercise condition, sampling time, study period, assigned sequence, exercise condition × time, period × time, and sequence × time were included as fixed effects. Participant and participant-specific study visit were included as random intercepts where supported by the model structure. If the visit-level random effect resulted in a singular fit, the model was simplified to a participant random intercept while retaining all prespecified fixed design factors.

The primary inferential parameter was the exercise condition × time interaction, representing the difference between conditions in the within-participant pre-to-post change: (Plantar flexion Post − Pre) − (Elbow flexion Post − Pre). Period and sequence were retained as design factors irrespective of their individual statistical significance. We estimated marginal means and contrasts using emmeans with Satterthwaite degrees of freedom. Unadjusted *p*-values for the ten primary exercise condition × time contrasts were corrected using the Holm procedure.

We also evaluated potential period- and sequence-related influences using the period × time and sequence × time terms. Complete outcome-specific estimates, 95% confidence intervals, unadjusted *p*-values, and Holm-adjusted *p*-values for these design-factor interactions are reported in Supplementary Table S3.

Because preliminary significance testing for carryover may be misleading, we did not use a formal carryover test to determine the primary analysis. Instead, we explored potential carryover using three complementary approaches. First, we summarized pre-exercise values separately by study period, sequence, and exercise condition (Supplementary Table S5A). Second, within-participant changes from the period-1 baseline to the period-2 baseline were summarized overall and by assigned sequence for each biomarker (Supplementary Table S5B). Third, we performed a first-period-only analysis as a sensitivity analysis because first-period observations cannot be affected by carryover from a preceding experimental condition; the complete results are reported in Supplementary Table S4.

We also performed a crossover-specific baseline-adjusted sensitivity analysis. For each participant, the plantar-minus-elbow post-exercise difference was modeled as a function of the corresponding plantar-minus-elbow pre-exercise difference and assigned sequence. This analysis evaluated whether conclusions based on the primary model were robust to between-visit differences in pre-exercise concentrations. We applied Holm correction separately across the ten biochemical outcomes within each sensitivity-analysis family.

Holm correction was applied across the ten exercise condition × time interaction tests. We calculated estimated marginal means and interaction contrasts using emmeans. The interaction effect size was calculated from the participant-level difference between condition-specific pre-to-post changes and expressed as Cohen’s d_z_ with a 95% confidence interval. Main effects of exercise condition and time were treated as secondary.

Diet, hydration, sleep, menstrual-cycle phase, and psychological stress were not included as quantitative covariates because they were not measured quantitatively.

The study was exploratory and systemic-profile-based. No single biochemical marker was designated as a standalone primary endpoint. The predefined biochemical panel included TC, HDL-C, LDL-C, TG, glucose, lactate, CK, LDH, GGT, and cortisol. We applied Holm correction across the family of exercise condition × time interaction tests to reduce type I error inflation. Statistical interpretation emphasized adjusted *p*-values, standardized interaction effect sizes, 95% confidence intervals, and the overall response pattern rather than isolated unadjusted findings. Main effects of exercise condition and time were considered secondary and descriptive.

The primary condition × time effect was evaluated using an explicit difference-in-differences contrast calculated from the model-estimated marginal means: (Plantar flexion Post − Pre) − (Elbow flexion Post − Pre). We obtained unadjusted *p*-values for each outcome-specific interaction contrast and subsequently adjusted them across the ten predefined biochemical outcomes using the Holm method. Cohen’s dz was calculated from the participant-level difference between the two condition-specific pre-to-post changes and is reported with a two-sided 95% confidence interval. We defined statistical significance as a Holm-adjusted *p*-value below 0.05.

We evaluated model assumptions using residual-versus-fitted plots, Q–Q plots, assessment of residual homoscedasticity, and standardized residual distributions. We conducted diagnostic assessments using the performance package.

Exercise-induced changes were additionally quantified as after–before deltas. To explore the multivariate structure of the biochemical response profile, we performed principal component analysis (PCA) on standardized complete-case delta values for the predefined biochemical panel.

PCA included 204 condition-specific change profiles from 102 participants. Because each participant contributed one profile per exercise condition, these observations were paired rather than statistically independent; PCA was therefore used only descriptively.

PCA was used as a descriptive visualization method rather than as an inferential test. Principal component scores were used to visualize the distribution of individual participants and exercise conditions in reduced-dimensional space, whereas loading vectors were used to identify which biochemical variables contributed most strongly to the observed multivariate pattern. No statistical significance testing was performed on PCA separation, and interpretation was based on visual overlap of scores, explained variance, and loading structure.

Because robust prior estimates of within-participant differences in systemic biochemical responses were unavailable, we performed a sensitivity analysis using the achieved sample size. The calculation was based on the paired difference-in-differences: D = (Post − Pre)_soleus-emphasizing condition_ − (Post − Pre)_biceps-emphasizing condition_.

With 102 participants, a two-sided α = 0.05, and 80% power, the minimum detectable standardized effect was d_z_ = 0.280. Because ten exercise condition × time interactions were included in the Holm-corrected primary family, a conservative sensitivity analysis using α = 0.005 was also performed, yielding a minimum detectable effect of d_z_ = 0.368. Thus, the study had reasonable sensitivity for moderate within-participant effects but limited sensitivity for small condition-dependent biomarker differences. The study was not adequately powered for definitive sex-specific or other subgroup analyses. These calculations characterize study sensitivity and do not retrospectively confirm nonsignificant findings.

Protocol-termination mode was summarized descriptively as objective task failure versus completion of the predefined 5 min cap. Because termination mode was determined during each exercise exposure and could differ between conditions within the same participant, no inferential subgroup analysis based on termination mode was included in the final analysis.

## 4. Results

### 4.1. Diagnostics and Cohort Characteristics

Residual diagnostics did not indicate major violations of model assumptions. Visual inspection of residual-versus-fitted and Q–Q plots showed acceptable homoscedasticity and approximate normality across outcomes. Diagnostic assessments conducted using the performance package did not indicate substantial residual heteroscedasticity, nonlinearity, or influential observations.

The plantar-flexion → elbow-flexion sequence comprised 19 men and 32 women, whereas the elbow-flexion → plantar-flexion sequence comprised 16 men and 35 women. All 102 participants completed both exercise conditions. Thus, the analysis-ready biochemical dataset used for the mixed-effects models contained complete biochemical observations for 102 participants. Table 1 presents descriptive characteristics for the full study cohort.

Because both exercise conditions frequently ended at the predefined 5 min cap rather than at objective task failure, we interpreted the biochemical analyses as responses to two standardized, localized exercise exposures rather than to two fully equivalent task-failure stimuli. This distinction is important because reaching the time cap indicates protocol termination, not objective biomechanical task failure.

Sex, anthropometric characteristics, and habitual physical-activity category are reported descriptively in Table 1, while the condition-specific protocol-termination mode is summarized in Table 2; no subgroup inferential analyses based on these variables were included.

Table 2 presents exercise-performance and task-demand characteristics. The conditions differed substantially in external load, anatomical region, and bilateral versus unilateral execution. Objective task failure occurred in 29 participants (28.5%) during the soleus-emphasizing condition and 22 participants (21.6%) during the biceps-brachii-emphasizing condition. The corresponding numbers reaching the predefined 5 min cap were 73 (71.5%) and 80 (78.4%), respectively. Consequently, the biochemical outcomes reflect responses to two non-equivalent standardized exercise exposures rather than matched objective exhaustion.

The plantar-flexion task consisted of bilateral seated calf raises performed with an external load of 10 kg for women and 20 kg for men, whereas the elbow-flexion task consisted of unilateral elbow flexion performed with 2 kg for women and 4 kg for men.

### 4.2. Crossover Design-Factor Analyses

The 102 participants were equally distributed between the two exercise sequences (*n* = 51 per sequence). After inclusion of study period and sequence in the mixed-effects models, none of the period × time or sequence × time effects remained statistically significant after Holm correction across the ten biochemical outcomes (Supplementary Table S3).

For CK, the period × time estimate was −36.639 U·L^−1^ (95% CI −91.212 to 17.935; *p* = 0.188) and the sequence × time estimate was −28.332 U·L^−1^ (95% CI −82.906 to 26.242; *p* = 0.308). For LDH, the corresponding estimates were −6.817 U·L^−1^ (95% CI −17.835 to 4.201; *p* = 0.225) and −5.938 U·L^−1^ (95% CI −16.956 to 5.080; *p* = 0.290), respectively. All corresponding Holm-adjusted *p*-values were 1.000. Complete period × time and sequence × time results for all ten biochemical outcomes are provided in Supplementary Table S3.

Supplementary Table S5A presents pre-exercise biochemical values stratified by study period, assigned sequence, and exercise condition. Within-participant period-2 minus period-1 changes in pre-exercise values, summarized overall and by assigned sequence, are presented in Supplementary Table S5B. Across all participants, the mean period-2 minus period-1 baseline shift was 18.51 ± 197.59 U·L^−1^ for CK and 4.66 ± 41.19 U·L^−1^ for LDH. These descriptive changes do not demonstrate systematic carryover; however, their substantial inter-individual variability and the 48–72 h inter-session interval mean that residual delayed enzyme effects cannot be excluded.

### 4.3. Main Exercise Condition × Time Interaction Effects

Period- and sequence-adjusted exercise condition × time interaction statistics for the predefined biochemical panel are presented in Table 3. Corresponding period- and sequence-adjusted estimated marginal means, condition-specific pre-to-post changes, and pre-exercise between-condition contrasts are presented in Supplementary Table S2.

For cortisol, the adjusted pre-exercise estimated marginal mean was 481.96 nmol·L^−1^ before the elbow-flexion condition and 424.06 nmol·L^−1^ before the plantar-flexion condition. The adjusted pre-exercise Plantar-minus-Elbow contrast was −57.90 nmol·L^−1^ (95% CI −119.33 to 3.54; *p* = 0.065; Supplementary Table S2). Thus, although the pre-exercise concentrations differed numerically between visits, the adjusted between-condition baseline contrast did not reach conventional statistical significance.

After Holm correction across the ten outcomes, cortisol remained the only statistically significant interaction. The adjusted cortisol interaction estimate was 149.219 nmol·L^−1^ (95% CI 62.338 to 236.099; unadjusted *p* = 0.000821; Holm-adjusted *p* = 0.008). The positive estimate indicates that the pre-to-post cortisol change was greater after the plantar-flexion task than after the elbow-flexion task. Because cortisol was measured only 2–3 min after task termination, this finding represents an early condition-dependent difference in change rather than a complete endocrine-response profile.

Glucose showed an adjusted negative exercise condition × time estimate of −7.013 mg·dL^−1^ (95% CI −12.238 to −1.788; unadjusted *p* = 0.009), indicating a more negative pre-to-post change following the plantar-flexion task. The primary interaction did not remain significant after correction across the ten outcomes (Holm-adjusted *p* = 0.078) and was therefore not considered statistically significant in the primary analysis. TC, HDL-C, LDL-C, TG, lactate, CK, LDH, and GGT likewise showed no statistically significant primary condition × time interactions after Holm correction. Because hemoglobin and hematocrit were not measured, none of the observed changes in circulating concentrations or enzyme activities were corrected for exercise-induced plasma-volume shifts; therefore, the magnitude of the reported early changes may partly reflect transient fluid redistribution.

TC, HDL-C, LDL-C, TG, lactate, CK, LDH, and GGT did not demonstrate statistically significant condition × time interactions after Holm correction. These nonsignificant results do not establish equivalence between conditions.

Supplementary Table S4 summarizes crossover-specific baseline-adjusted and first-period-only sensitivity analyses for all ten outcomes. For cortisol, the crossover baseline-adjusted analysis yielded an estimate of 87.784 nmol·L^−1^ (95% CI 25.690 to 149.879; *p* = 0.006), which narrowly failed to remain significant after correction across the ten sensitivity-analysis outcomes (Holm-adjusted *p* = 0.054). The first-period-only cortisol estimate was 67.765 nmol·L^−1^ (95% CI −59.692 to 195.222; *p* = 0.294). Thus, although the cortisol interaction remained statistically significant in the prespecified period- and sequence-adjusted primary model, its magnitude and statistical support were less consistent in alternative crossover analyses.

Conversely, glucose showed a significant effect in the crossover baseline-adjusted sensitivity analysis (−8.918 mg·dL^−1^, 95% CI −12.406 to −5.430; Holm-adjusted *p* = 0.000018) and in the first-period-only analysis (−4.989 mg·dL^−1^, 95% CI −8.306 to −1.671; Holm-adjusted *p* = 0.036). These findings support a reproducible glucose signal across alternative analytical approaches, although glucose did not meet the multiplicity-corrected significance criterion in the prespecified primary mixed-effects model.

### 4.4. Lipid-Related Outcomes

**Figure 3:**
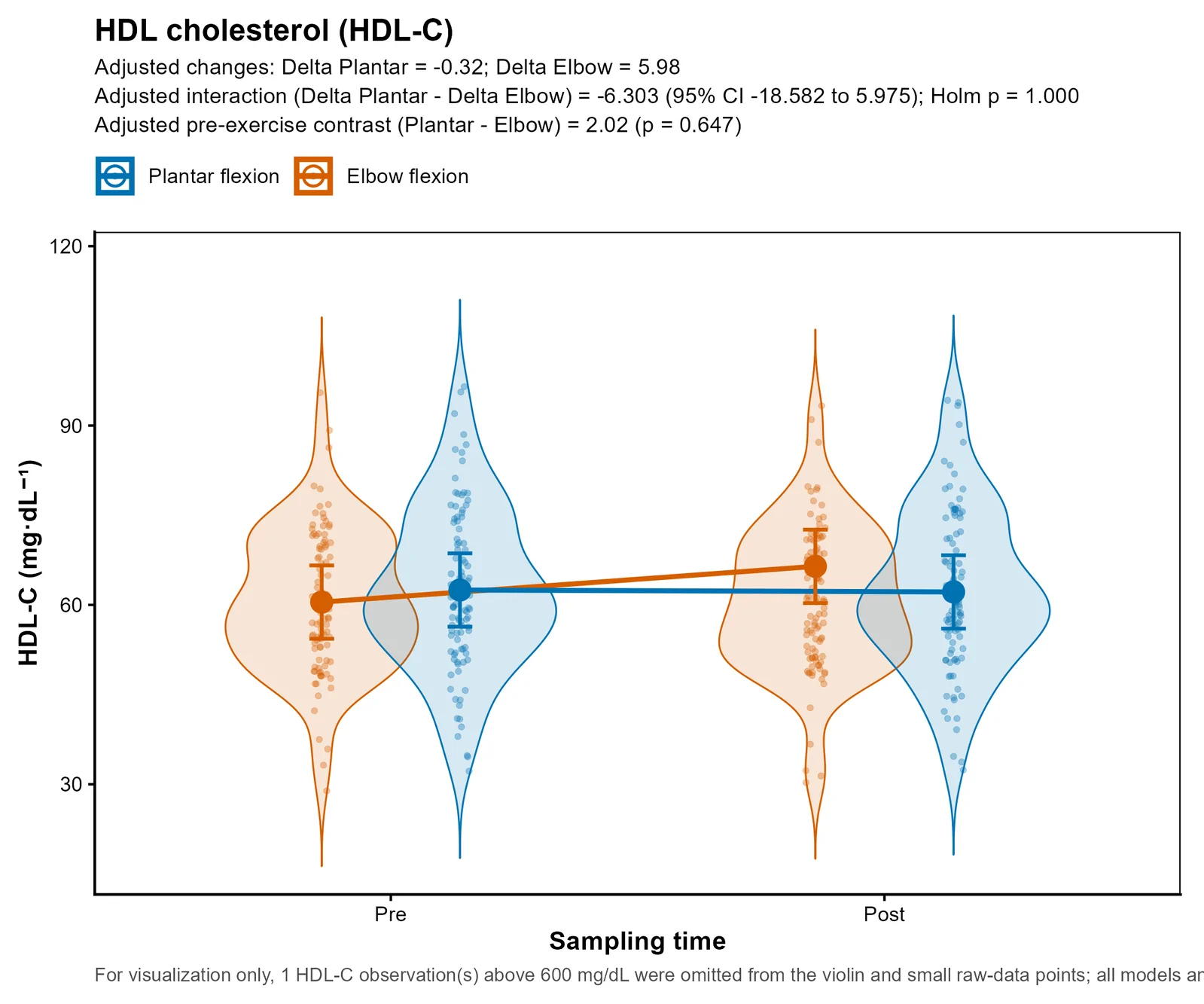
High-density lipoprotein cholesterol (HDL-C) concentrations before and after the plantar-flexion task and elbow-flexion task. Values are expressed in mg·dL^−1^. The model-based estimates shown in the figure were obtained from mixed-effects models adjusted for study period and assigned sequence.

**Figure 4:**
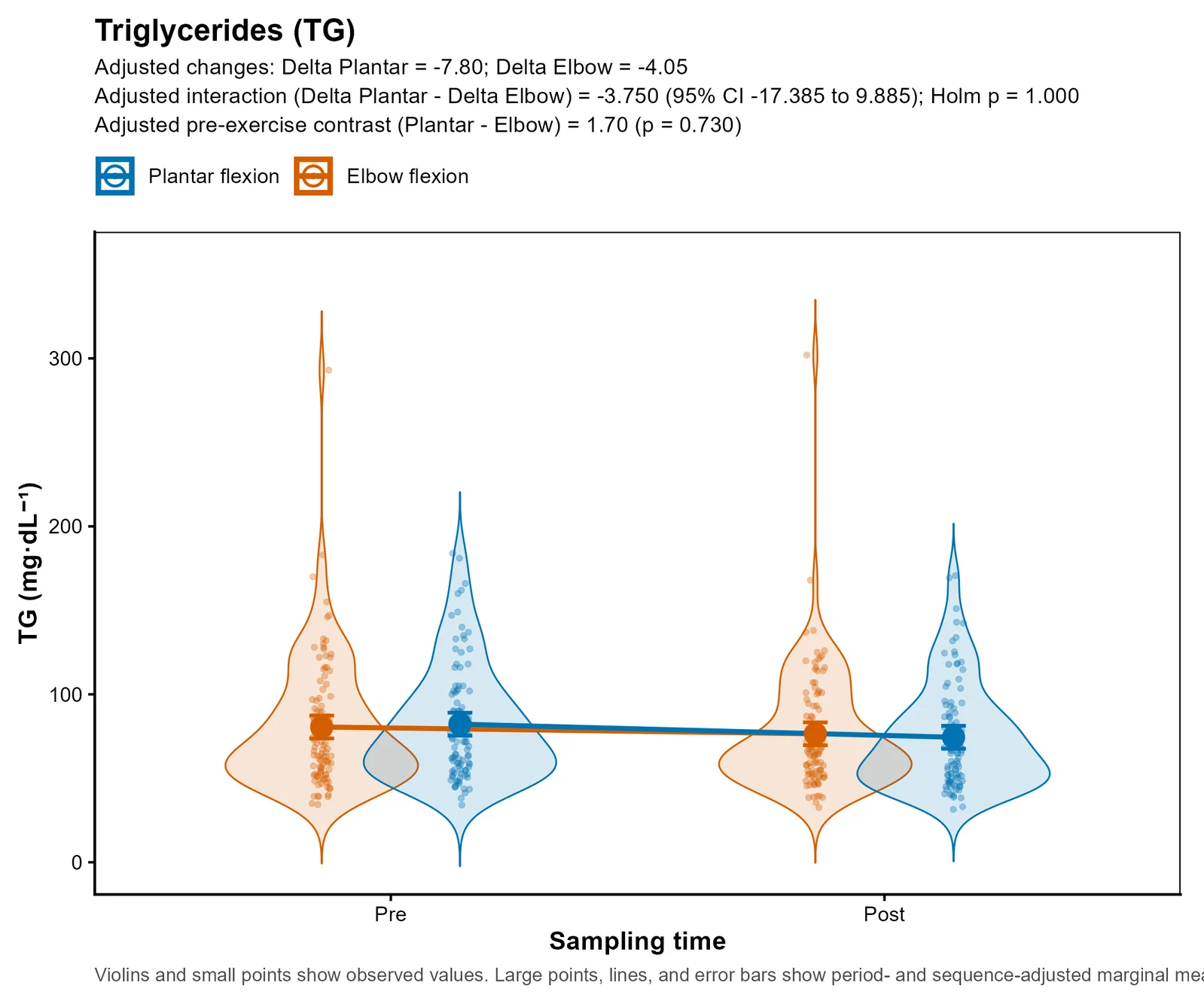
Triglyceride (TG) concentrations before and after the plantar-flexion task and elbow-flexion task. Values are expressed in mg·dL^−1^. The model-based estimates shown in the figure were obtained from mixed-effects models adjusted for study period and assigned sequence.

**Figure 5:**
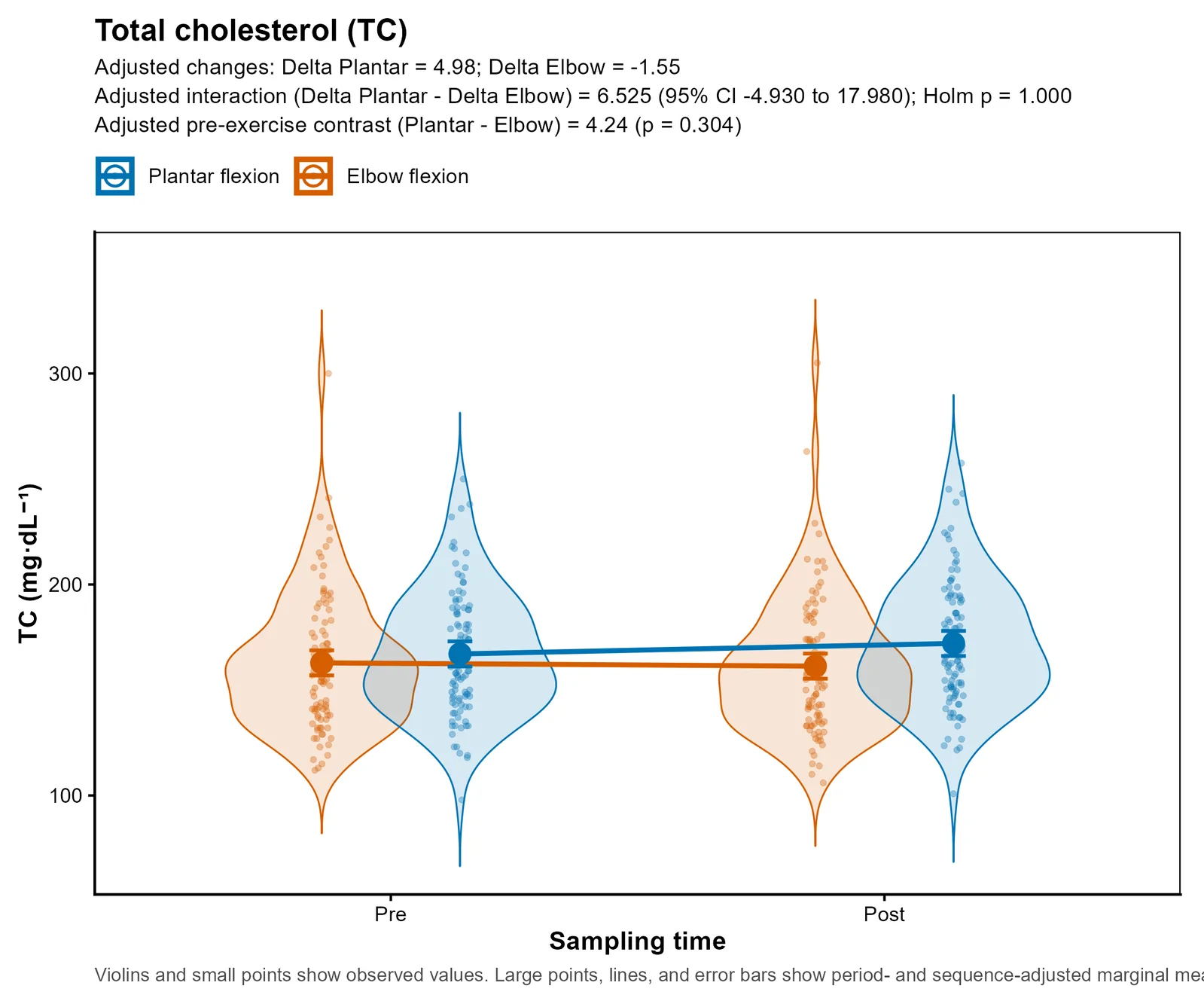
Total cholesterol (TC) concentrations before and after the plantar-flexion task and elbow-flexion task. Values are expressed in mg·dL^−1^. The model-based estimates shown in the figure were obtained from mixed-effects models adjusted for study period and assigned sequence.

**Figure 6:**
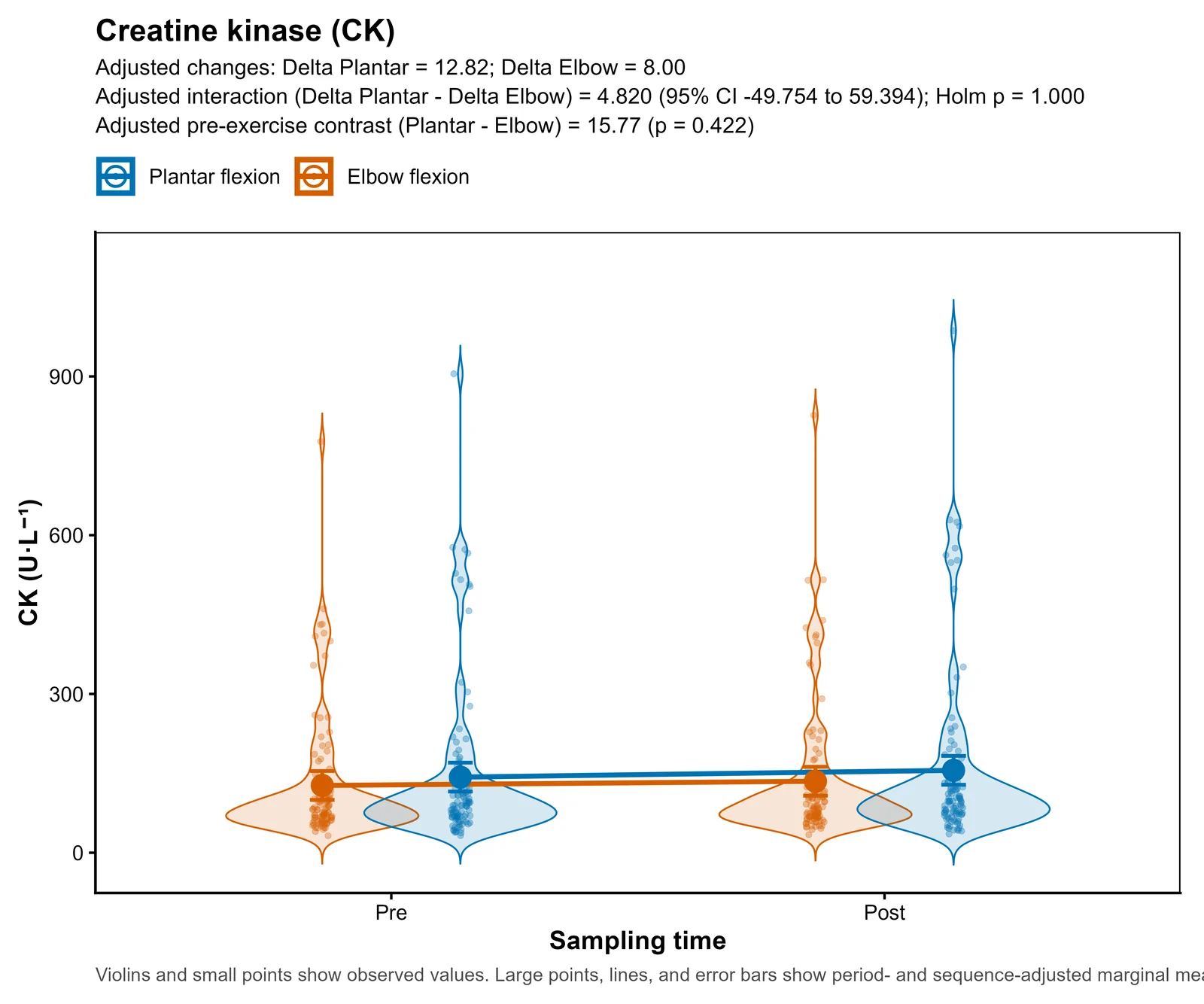
Creatine kinase (CK) activity before and after the plantar-flexion task and elbow-flexion task. Values are expressed in U·L^−1^. The model-based estimates shown in the figure were obtained from mixed-effects models adjusted for study period and assigned sequence.

Model-estimated marginal means for TC, HDL-C, LDL-C, and TG are presented in Figure 3, Figure 4, Figure 5 and Figure 6. None of the lipid-related outcomes demonstrated statistically significant exercise condition × time interactions after Holm correction. TC, HDL-C, LDL-C, and TG all showed confidence intervals crossing zero and small standardized interaction effects. Although TG and HDL-C showed numerically greater post-exercise reductions following the soleus-emphasizing plantar-flexion condition than the biceps-brachii-emphasizing elbow-flexion condition, these differences remained nonsignificant after multiplicity correction. Therefore, lipid-related outcomes did not demonstrate statistically robust separation between exercise conditions.

### 4.5. Enzymatic Outcomes

**Figure 7:**
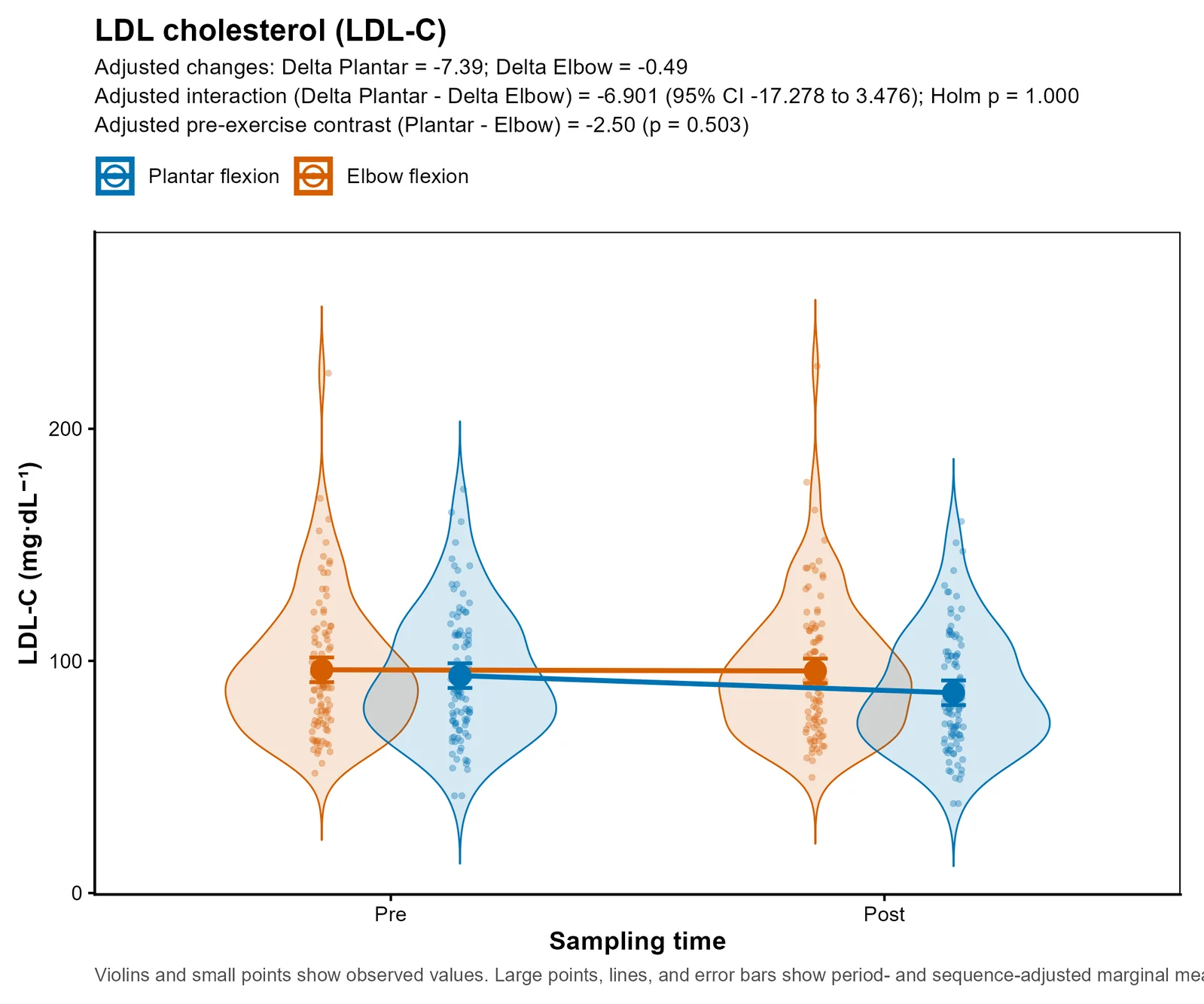
Low-density lipoprotein cholesterol (LDL-C) concentrations before and after the plantar-flexion task and elbow-flexion task. Values are expressed in mg·dL^−1^. The model-based estimates shown in the figure were obtained from mixed-effects models adjusted for study period and assigned sequence.

**Figure 8:**
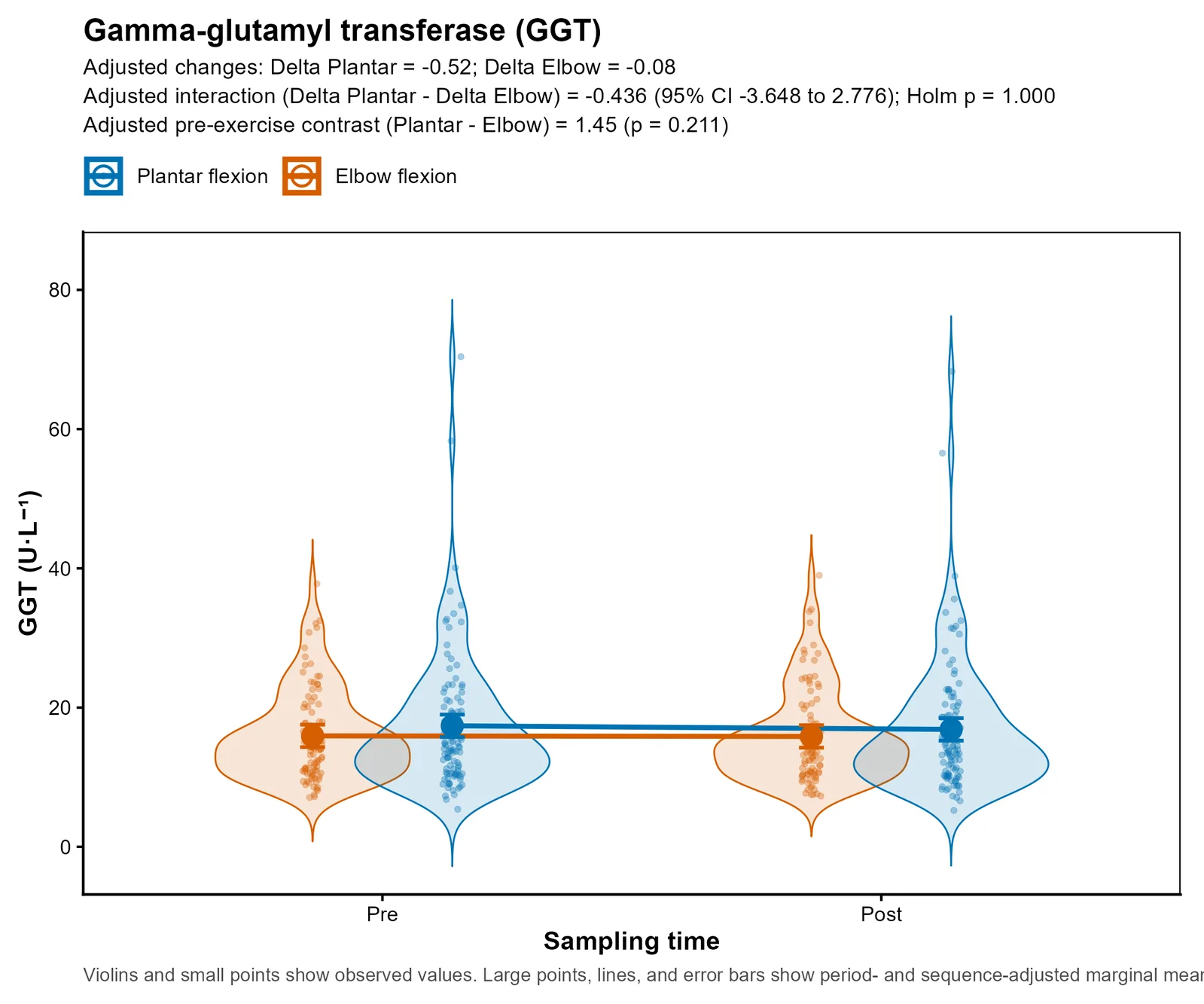
γ-Glutamyl transferase (GGT) activity before and after the plantar-flexion task and elbow-flexion task. Values are expressed in U·L^−1^. The model-based estimates shown in the figure were obtained from mixed-effects models adjusted for study period and assigned sequence.

Figure 7 and Figure 8 show changes in LDH and CK, respectively. LDH and CK increased numerically after both exercise conditions, with slightly larger displayed changes following the soleus-emphasizing condition. However, neither condition × time interaction was statistically significant after Holm correction. Because blood was collected only 2–3 min after exercise, these findings reflect very early changes in circulating CK and LDH activity and do not characterize their delayed kinetics or assess exercise-induced muscle damage.

### 4.6. Hormonal and Metabolic Outcomes

**Figure 9:**
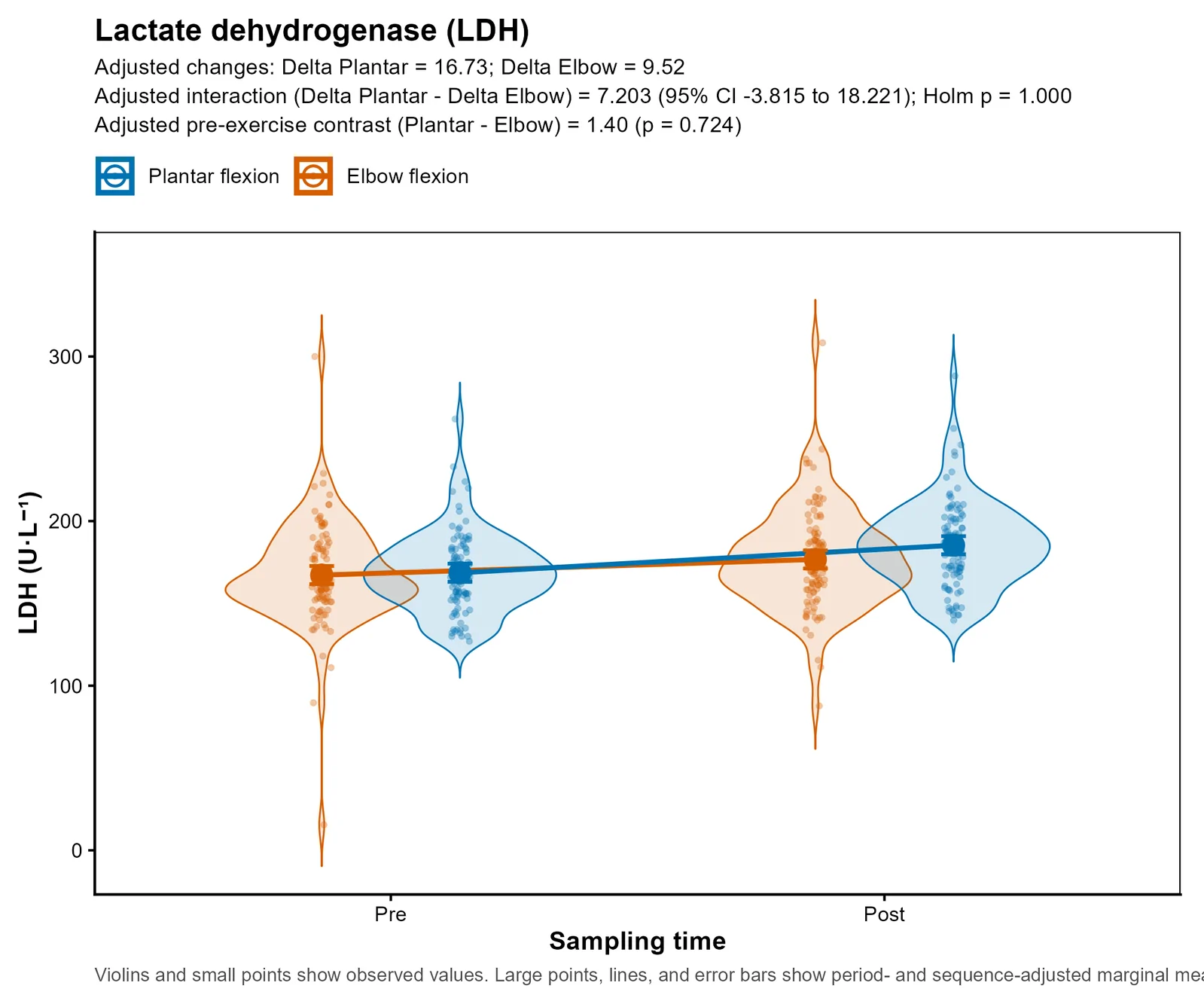
Lactate dehydrogenase (LDH) activity before and after the plantar-flexion task and elbow-flexion task. Values are expressed in U·L^−1^. The model-based estimates shown in the figure were obtained from mixed-effects models adjusted for study period and assigned sequence.

**Figure 10:**
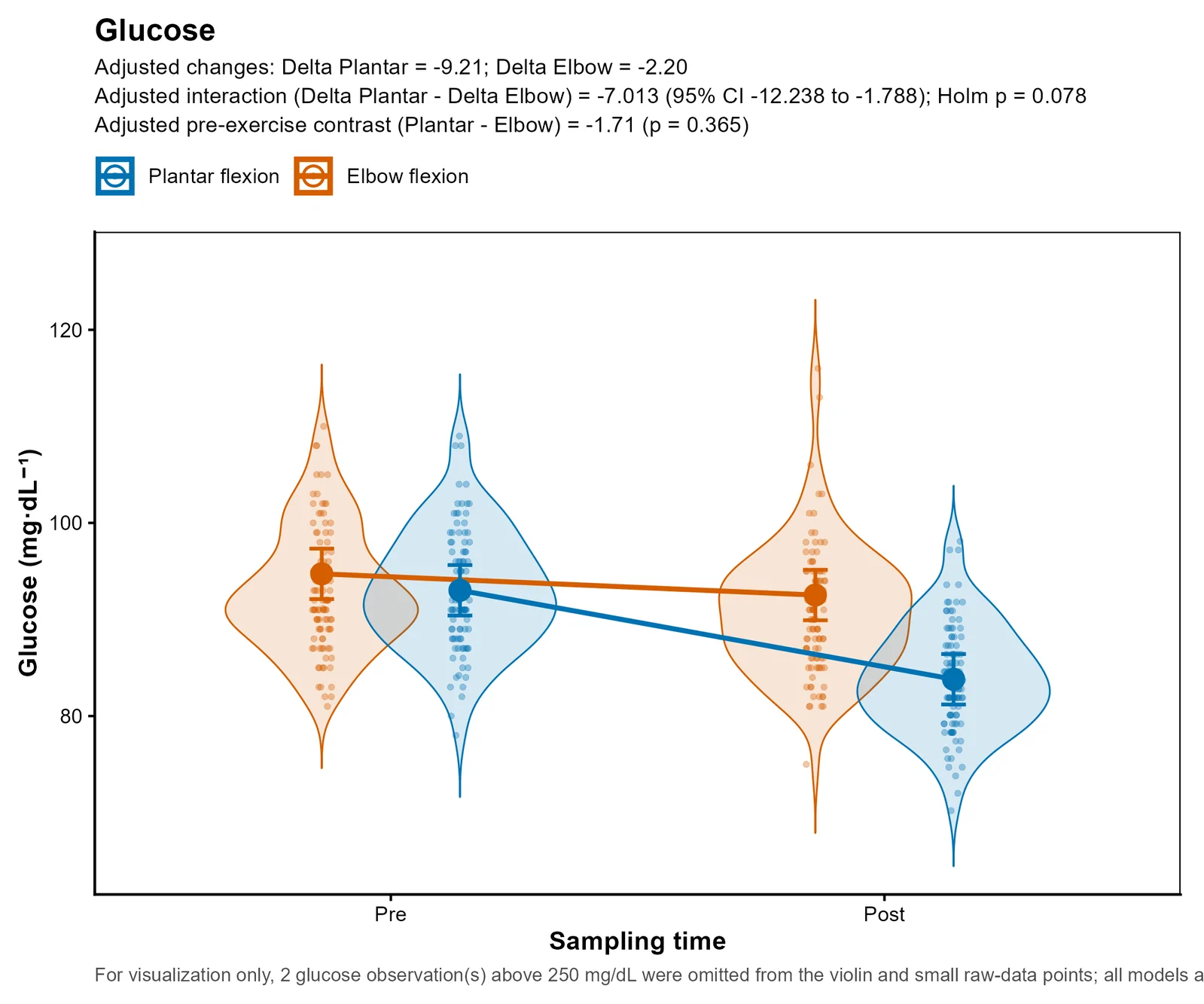
Plasma glucose concentrations before and after the plantar-flexion task and elbow-flexion task. Values are expressed in mg·dL^−1^. The model-based estimates shown in the figure were obtained from mixed-effects models adjusted for study period and assigned sequence.

**Figure 11:**
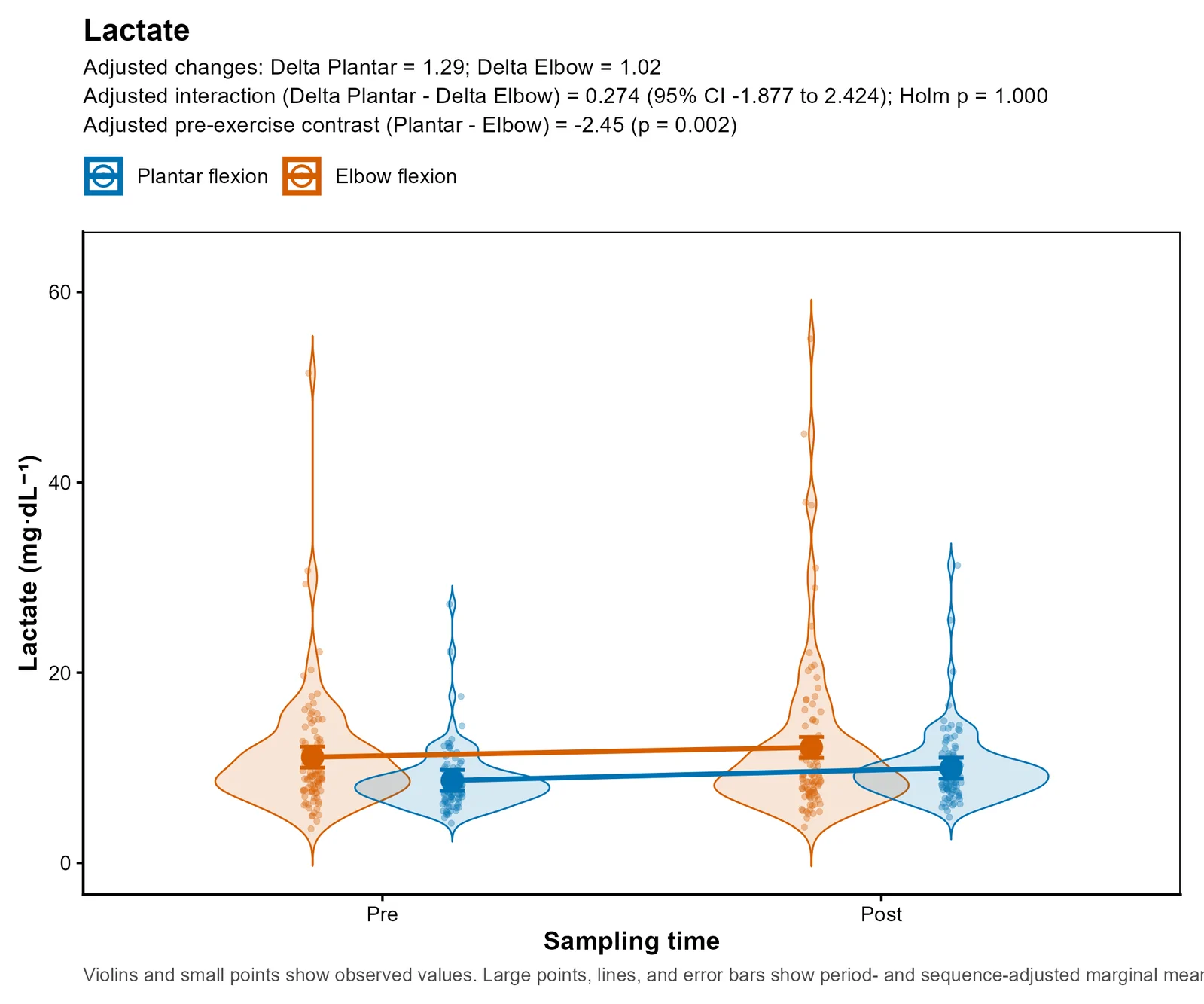
Plasma lactate concentrations before and after the plantar-flexion task and elbow-flexion task. Values are expressed in mg·dL^−1^. The model-based estimates shown in the figure were obtained from mixed-effects models adjusted for study period and assigned sequence.

**Figure 12:**
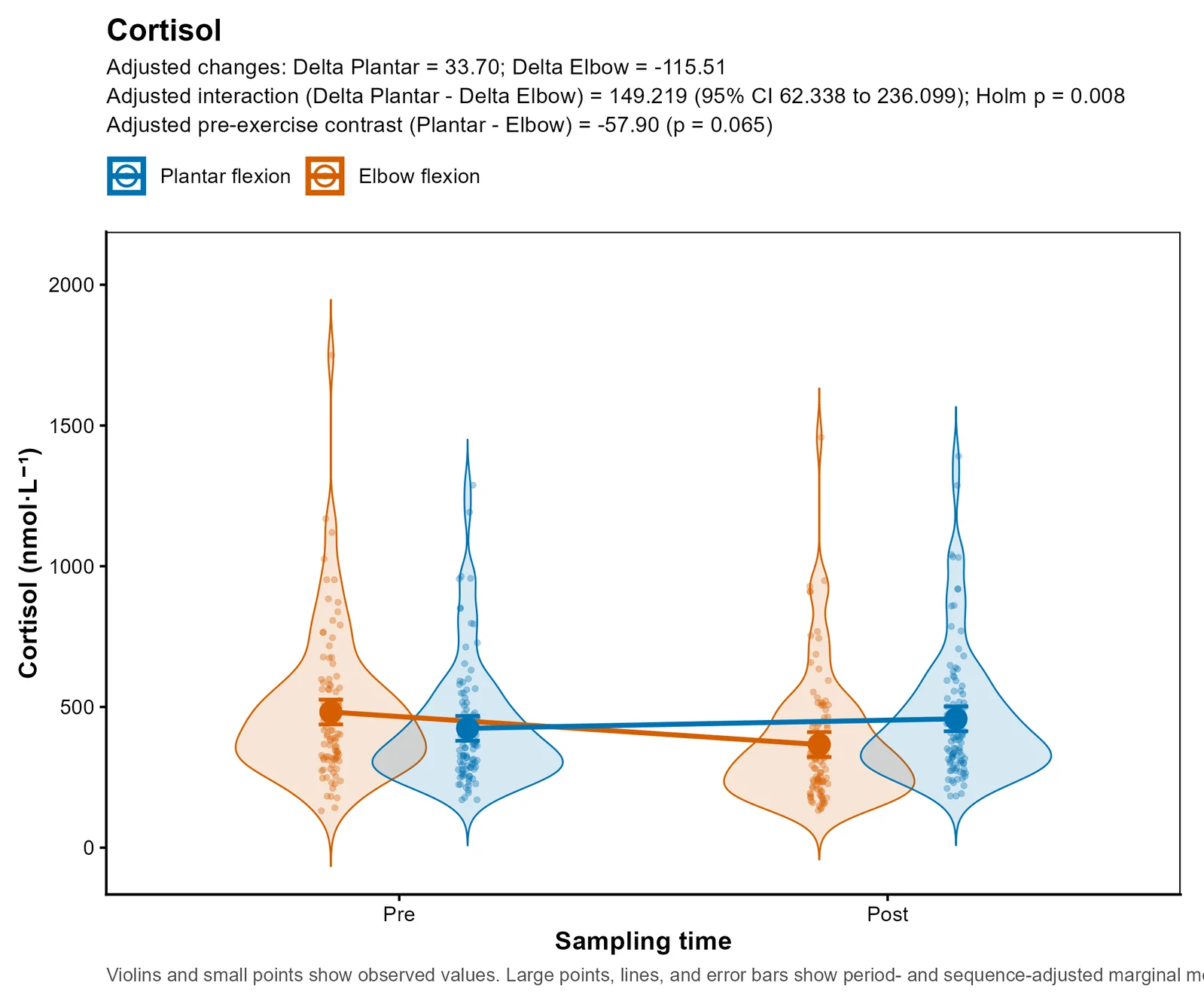
Serum cortisol concentrations before and after the plantar-flexion task and elbow-flexion task. Values are expressed in nmol·L^−1^. The model-based estimates shown in the figure were obtained from mixed-effects models adjusted for study period and assigned sequence.

Changes in glucose, lactate, cortisol, and GGT are shown in Figure 9, Figure 10, Figure 11 and Figure 12. In the period- and sequence-adjusted primary models, cortisol demonstrated a statistically significant exercise condition × time interaction after Holm correction (149.219 nmol·L^−1^, 95% CI 62.338 to 236.099; unadjusted *p* = 0.000821; Holm-adjusted *p* = 0.008). Glucose showed a negative interaction estimate of −7.013 mg·dL^−1^ (95% CI −12.238 to −1.788; unadjusted *p* = 0.009), but it was not statistically significant after Holm correction (adjusted *p* = 0.078). Lactate and GGT showed no statistically significant adjusted interactions. Complete adjusted marginal means and condition-specific changes are provided in Supplementary Table S2, and crossover sensitivity analyses are presented in Supplementary Table S4.

Lactate did not show a statistically significant exercise condition × time interaction after multiple-testing correction. However, this should not be interpreted as evidence of equivalent glycolytic responses to the two plantar flexion and elbow flexion tasks, because both tasks frequently ended by time cap and the biceps-brachii-emphasizing elbow-flexion task involved a smaller unilateral upper-limb exposure with lower absolute external load. GGT also showed no significant interaction effect. Because cortisol was measured only 2–3 min after exercise, the significant interaction should be interpreted specifically as an early condition-dependent difference in the pre-to-post change in circulating cortisol concentration, rather than as evidence of peak HPA-axis activation or a complete endocrine-response profile.

### 4.7. Principal Component Analysis

**Figure 13:**
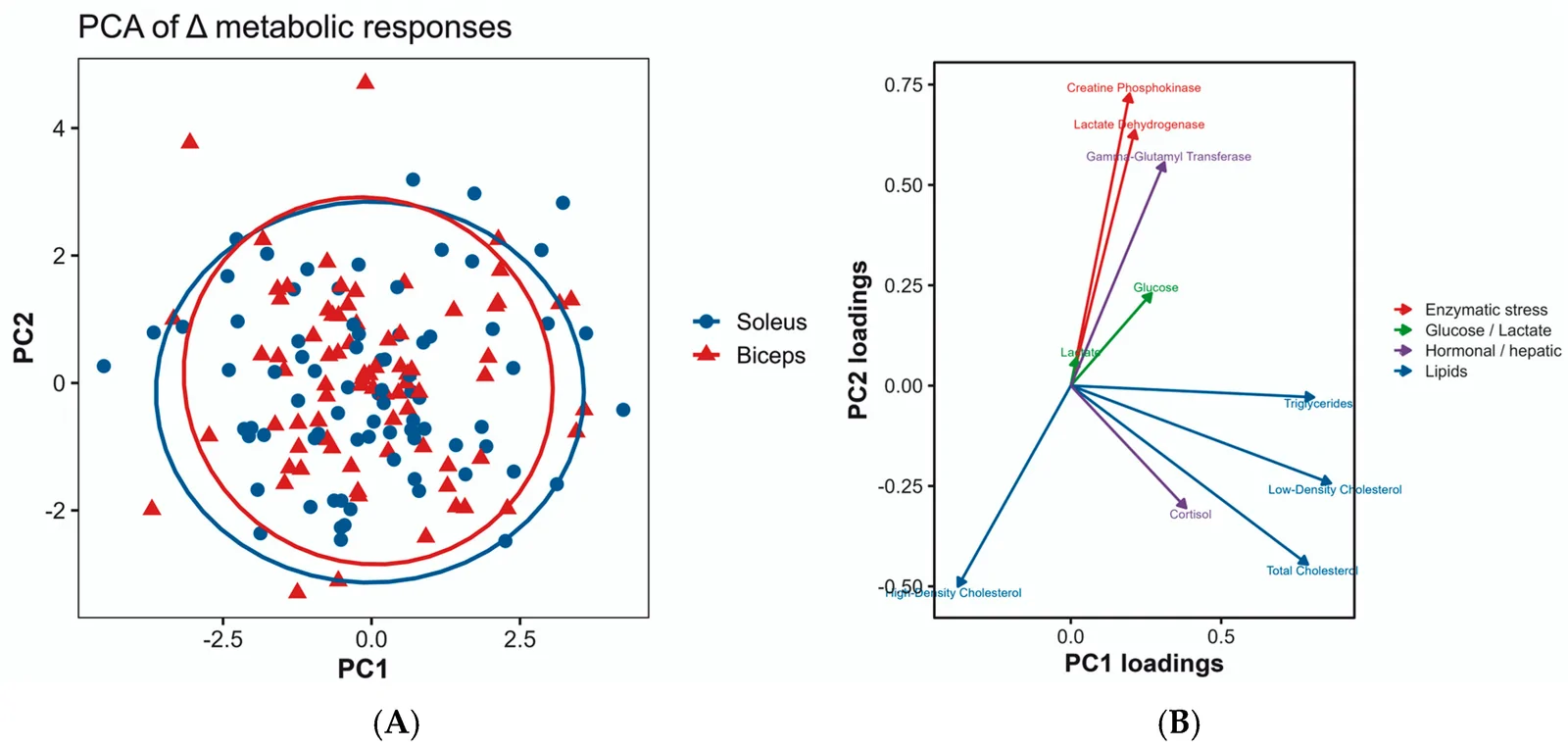
Principal component analysis of 204 standardized condition-specific biochemical change profiles from 102 participants. (**A**) Score plot for the plantar-flexion and elbow-flexion exercise conditions. Ellipses represent 95% concentration ellipses and are shown only to facilitate descriptive visualization; they should not be interpreted as confidence regions or as inferential evidence of group separation. (**B**) Loading plot showing the contributions of the individual biomarkers. PC1 and PC2 explained 24.9% and 17.5% of the total variance, respectively.

Principal component analysis was used descriptively to visualize covariance among standardized biochemical change scores. Beyond the biomarker-specific analyses, PCA was used only to visualize the dominant covariance structure among simultaneous biochemical changes and to identify which variables contributed most strongly to the principal axes of variation. PC1 and PC2 explained 24.9% and 17.5% of the total variance, respectively. The score plot showed substantial visual overlap between conditions (Figure 13A,B). Because PCA maximizes overall variance rather than between-condition discrimination, does not formally account for the paired structure in this application, and was not followed by an inferential test of separation, this overlap should not be interpreted as evidence of multivariate equivalence between conditions.

## 5. Discussion

The principal finding of this exploratory within-participant crossover study was a statistically significant condition-dependent difference in the pre-to-early-post cortisol change after Holm correction. Glucose showed a nominal condition × time interaction, but this finding did not remain statistically significant after correction across the ten outcomes. The lipid-related, lactate, enzymatic, and GGT interaction tests were also nonsignificant after Holm correction. The first two PCA dimensions showed substantial visual overlap between conditions; however, PCA was used descriptively and did not constitute an inferential test of paired multivariate separation or equivalence. Because most participants reached the predefined 5 min cap rather than objective task failure, the findings describe responses to two standardized but non-equivalent exercise exposures rather than responses to uniform exhaustion.

Human movement is generated through coordinated activation of agonists, synergists, stabilizers, and, in some circumstances, antagonists. Although knee flexion was used to reduce the mechanical contribution of the gastrocnemius during seated plantar flexion, it did not eliminate gastrocnemius or other plantar-flexor activity. Similarly, elbow flexion could not isolate the biceps brachii from the brachialis, brachioradialis, shoulder stabilizers, and antagonist coactivation. Because EMG or another direct measure of recruitment was not recorded, the relative contribution and fatigue of individual muscles remain unknown. Consequently, the present results describe systemic responses to two multi-muscle plantar flexion and elbow flexion tasks and cannot establish muscle-specific or fiber-type-specific biochemical effects.

The exploratory nature of the study is important for interpretation. The biomarker panel was intended to characterize systemic biochemical response patterns rather than to test a single confirmatory biochemical endpoint. Therefore, the significant cortisol interaction and the nominal glucose interaction should be interpreted as exploratory, condition-dependent systemic responses. In contrast, the absence of significant interactions for the remaining biomarkers should be interpreted as a lack of detectable separation across most of the selected systemic profile, not as definitive confirmation or rejection of a specific mechanistic pathway.

The cortisol interaction requires particular caution. The period- and sequence-adjusted pre-exercise estimated marginal mean was 481.96 nmol·L^−1^ before the elbow-flexion condition and 424.06 nmol·L^−1^ before the plantar-flexion condition, corresponding to an adjusted Plantar-minus-Elbow baseline contrast of −57.90 nmol·L^−1^ (95% CI −119.33 to 3.54; *p* = 0.065; Supplementary Table S2). Although this contrast did not reach conventional statistical significance, the numerical difference indicates that visit-related factors cannot be excluded. Because these measurements preceded exercise, such variation may reflect anticipatory or procedural stress, residual circadian or visit-specific influences, or regression to the mean rather than an effect of the subsequently performed task. Importantly, the cortisol condition × time interaction remained statistically significant in the primary model after adjustment for period and sequence (149.219 nmol·L^−1^; Holm-adjusted *p* = 0.008), but support was less consistent in the alternative crossover analyses: the baseline-adjusted sensitivity analysis yielded a Holm-adjusted *p* = 0.054, and the first-period-only analysis was not statistically significant (Supplementary Table S4). Because blood was collected only 2–3 min after task termination, the finding should therefore be interpreted as an early condition-dependent difference in change rather than evidence of a task-specific endocrine profile or a complete cortisol-response pattern.

A key interpretive constraint is that the protocol does not isolate fiber-type composition as an independent experimental variable. The soleus and biceps brachii differ not only in their oxidative profiles and fiber-type distributions but also in anatomical location, muscle mass, architecture, habitual recruitment, vascular supply, bilateral versus unilateral activation, external load, mechanical leverage, and task characteristics. Therefore, any observed differences, or lack of differences, cannot be attributed solely to muscle fiber composition. The findings should instead be interpreted as systemic biochemical responses to two functionally divergent localized exercise conditions.

Accordingly, any observed condition-dependent effect identifies a difference between the two complete plantar flexion and elbow flexions and should not be interpreted as evidence that muscle functional phenotype itself caused that difference. Alternative explanations include differences in active muscle mass, anatomical region, bilateral versus unilateral execution, relative intensity, external loading, total exercise demand, perceived effort, and proximity to task failure.

Although objective task-failure criteria were defined for both tasks, most participants reached the predefined 5 min cap before objective task failure occurred. Therefore, proximity to exhaustion likely differed between participants and between exercise conditions. This issue is particularly relevant for the biceps-brachii-emphasizing condition, which involved a smaller unilateral upper-limb task and lower absolute external load. Consequently, the applied elbow-flexion task may not have consistently produced sufficient fatigue or strain, and the absence of larger biochemical responses should not be interpreted as evidence of absent glycolytic or mechanical potential.

Under the present protocol and biomarker panel, detectable condition-dependent systemic effects appeared limited. Nonetheless, model-estimated marginal means showed directionally consistent differences for selected outcomes, particularly triglycerides and glucose, although these patterns should be interpreted cautiously because most outcomes did not remain significant after correction, and the plantar flexion and elbow flexions were not physiologically equivalent.

Sensitivity was subsequently characterized using the achieved analytic sample. Under a paired-test approximation, *N* = 102, two-sided, a = 0.05, and 80% power corresponded to a minimum detectable standardized difference-of-differences of d_z_ = 0.280. Using a = 0.005 as a conservative approximation for the ten-outcome family increased the minimum detectable effect to d_z_ = 0.368. These calculations indicate limited sensitivity to small effects and do not establish equivalence or retrospectively confirm null findings.

The crossover design controlled stable participant characteristics because every participant completed both conditions. Participants followed a standardized meal list before and during the experimental period, which was intended to reduce between-session variation in short-term dietary intake. Nevertheless, meals were not weighed, and energy and macronutrient intakes were not quantitatively verified. Visit-specific variation in hydration, sleep, psychological stress, recent activity, and menstrual-cycle-related physiology may also have contributed to residual within-participant variability. The dietary, fasting, caffeine, alcohol, and exercise instructions reduced but did not eliminate these potential influences.

Most nonsignificant interaction estimates were smaller than the conservatively estimated detectable effect. The study therefore cannot exclude small condition-dependent differences in these outcomes. The nonsignificant results should be interpreted as effects that were small or imprecisely estimated under the present protocol, not as evidence of equivalence. No equivalence margins were prespecified, and no formal equivalence tests were performed. Consequently, nonsignificant interactions indicate that the present analysis did not demonstrate a difference; they do not establish equivalence between conditions.

As shown in Table 3, the negative glucose interaction estimate indicates a more negative estimated pre-to-post change following the soleus-emphasizing condition than following the biceps-brachii-emphasizing condition; however, this interaction did not remain statistically significant after Holm correction. These patterns are best viewed as exploratory and hypothesis-generating because circulating biomarkers cannot isolate intramuscular substrate oxidation, and the plantar flexion and elbow flexions were not mechanically or physiologically equivalent.

Several factors may have contributed to the absence of significant exercise condition × time interactions for most biochemical outcomes. These include insufficient or non-equivalent exercise stimulus, heterogeneous workload between the two tasks, high inter-individual variability, the immediate timing of post-exercise blood sampling, and the limited specificity of circulating biomarkers for localized muscle metabolism. Consequently, the null findings should not be interpreted as evidence that the two plantar flexion and elbow flexions produced equivalent physiological responses. Rather, they indicate that differences between the applied plantar-flexion and elbow-flexion tasks were not detectable for most circulating biomarkers under the present protocol.

### 5.1. Systemic Lipid-Related Responses to the Two Plantar Flexion and Elbow Flexion

Circulating lipid concentrations reflect the integrated balance between hepatic lipoprotein production, peripheral tissue uptake, lipolytic activity, and plasma volume dynamics. Although oxidative muscles are known to rely more on lipid metabolism during sustained activity, the present study did not directly assess tissue-specific lipid uptake or oxidation.

Therefore, although minor directional differences in triglyceride responses were observed, these cannot be attributed to specific intramuscular mechanisms. Under the two non-equivalent protocols, we observed no statistically detectable differential change in circulating lipid concentrations. This finding does not establish equivalence or a muscle-specific null effect.

The modest reduction in HDL-C observed after the soleus-emphasizing condition should also be interpreted cautiously, as acute HDL-C changes may reflect transient plasma volume shifts and lipoprotein remodeling. LPL-mediated hydrolysis alters HDL particle composition by transferring surface lipids and apolipoproteins from triglyceride-rich lipoproteins [21], and short-term HDL-C reductions may therefore reflect dynamic lipid redistribution rather than impaired HDL function.

### 5.2. Glucose Handling Pathways

As shown in Table 3, the negative glucose interaction estimate indicates a more negative estimated pre-to-post change after the plantar-flexion task than after the elbow-flexion task. However, this interaction did not remain statistically significant after Holm correction and should therefore be interpreted as a nominal, hypothesis-generating pattern. The observed difference may reflect total active muscle mass, bilateral versus unilateral performance, relative intensity, hepatic output, or other whole-body processes. Because local glucose uptake, muscle recruitment, blood flow, and substrate oxidation were not measured, this interaction cannot be attributed specifically to soleus metabolism or fiber-type composition.

### 5.3. Interpretation of Lactate and Oxidative Metabolism-Related Responses

The absence of a significant lactate interaction should be interpreted cautiously. Blood lactate reflects the balance between local production, release into circulation, distribution volume, oxidation, and clearance. Because lactate was measured only once, 2–3 min after exercise, and because the two tasks differed in workload, muscle mass, and proximity to task failure, the present data cannot determine whether intramuscular glycolytic flux differed between conditions. The significant cortisol interaction represents a condition-dependent difference in the pre-to-early-post cortisol change. However, because cortisol was sampled only 2–3 min after exercise, this finding should not be interpreted as a complete characterization of hypothalamic–pituitary–adrenal activation or recovery kinetics [22].

### 5.4. Enzymatic Markers

The CK and LDH findings, as well as the condition-dependent cortisol finding, should be interpreted in light of the single early post-exercise sampling window.

Blood was collected within 2–3 min after task termination. This interval represents an early recovery time point but may precede the peak blood-lactate response and is insufficient to capture the delayed kinetics of CK and LDH or the full temporal profile of cortisol. CK and LDH may increase hours after exercise, particularly after damaging, eccentric, or high-volume contractions, whereas cortisol responses depend on exercise intensity, duration, psychological stress, circadian rhythm, and recovery dynamics. Therefore, the CK and LDH measurements should be interpreted only as very early changes in circulating enzyme activity and not as indicators of the presence or absence of exercise-induced muscle damage. No statistically distinguishable very early CK or LDH response was detected between plantar flexion and elbow flexion. Similarly, the cortisol measurement represents only an early circulating response and cannot characterize the complete endocrine-response profile. Accordingly, the 2–3 min sampling window is informative for characterizing the immediate circulating response to plantar flexion and elbow flexion, but not for evaluating delayed muscle-damage-associated enzyme release.

The crossover analyses provided no statistically significant evidence that the immediate pre-to-post CK or LDH responses differed systematically by study period or assigned sequence. For CK, neither the period × time term nor the sequence × time term was significant; the same was true for LDH (Supplementary Table S3). Nevertheless, descriptive period-2 minus period-1 baseline shifts averaged 18.51 U·L^−1^ for CK and 4.66 U·L^−1^ for LDH, with substantial inter-individual variability, particularly for CK (Supplementary Table S5B). These observations do not establish a carryover effect, but they also do not exclude residual delayed enzyme activity in individual participants. Accordingly, the 48–72 h inter-session interval remains a limitation for biomarkers with delayed post-exercise kinetics.

### 5.5. Integrated Multivariate Perspective

The descriptive PCA score plot showed substantial visual overlap between conditions in the first two principal components. This visualization suggests that between-participant variation was prominent relative to the visible condition-related structure in these components. However, PCA was not an inferential test of paired multivariate differences, and the observed overlap does not establish equality or equivalence of the complete biochemical response profiles.

### 5.6. Systemic Biomarkers Versus Direct Intramuscular Methods

A central methodological consideration is that circulating biochemical markers are not substitutes for direct intramuscular measurements. Plasma glucose, triglycerides, lactate, CK, LDH, GGT, and cortisol reflect integrated whole-body regulation, including skeletal muscle uptake, hepatic glucose production, adipose tissue lipolysis, endocrine regulation, plasma volume shifts, clearance kinetics, and inter-individual variability. Because plasma-volume change was not measured, the immediate pre-to-post changes observed in circulating concentrations and enzyme activities may partly reflect transient fluid redistribution, in addition to changes in production, uptake, release, or clearance. Therefore, these markers cannot determine muscle fiber composition, quantify substrate oxidation within a specific muscle, or directly assess mitochondrial or enzymatic activity in the activated tissue. Such mechanistic questions would require direct or localized approaches, including muscle biopsy, intramuscular assessment of glycogen or lipids, stable isotope tracers, near-infrared spectroscopy, local blood-flow assessment, direct measurement of muscle recruitment, serial metabolomics, or direct mitochondrial and enzymatic assays.

Nevertheless, systemic biochemical markers have practical and translational relevance. Invasive or technically demanding methods such as biopsy, microdialysis, stable-isotope tracing, or ex vivo enzymatic assays are valuable for mechanistic studies. However, they are not practical for routine training prescriptions, rehabilitation monitoring, public health exercise guidance, or body-composition-oriented exercise selection. The present study therefore addresses whether accessible circulating biomarkers can detect systemic biochemical changes following specific localized plantar flexion and elbow flexion. The findings show that selected condition-dependent changes can be detected, but the study does not establish the validity or practical utility of these biomarkers for monitoring localized muscle-specific responses.

### 5.7. Physiological and Translational Implications

A practical implication of this study is that routine circulating biomarkers should be interpreted cautiously after localized exercise because they cannot identify the contribution of an individual muscle or distinguish local substrate use from integrated whole-body regulation. In practical settings, circulating biomarkers may help characterize integrated systemic responses to localized exercise, but they should not be used to infer tissue-specific substrate oxidation, fiber-type recruitment, or mitochondrial function. Whether similar systemic responses occur in older adults, individuals with metabolic disorders, or patients undergoing early rehabilitation remains to be determined, and this question should be addressed in future studies specifically designed for these populations.

A specific limitation of the present protocol is that the biceps-brachii-emphasizing condition included a predefined 5 min time cap. This cap was introduced to prevent the task from becoming a prolonged low-intensity endurance activity and to preserve the intended resistance-type character of the exercise. However, because most participants reached this cap before objective biomechanical task failure, the biceps condition should not be interpreted as a uniform task-failure stimulus. Instead, it should be regarded as a standardized localized upper-limb exercise exposure with variable proximity to true fatigue.

This issue is important when interpreting the metabolic and enzymatic outcomes. Because the conditions were unmatched for active muscle mass, laterality, relative intensity, external load, and proximity to task failure, the study cannot determine whether the biceps-brachii-emphasizing condition would have produced larger lactate, CK, or LDH responses under physiologically matched conditions. Therefore, the absence of larger lactate, CK, or LDH responses after the elbow-flexion task may reflect a lower effective stimulus produced by that task rather than a true absence of muscle-specific metabolic differences.

Accordingly, the present findings should be interpreted as responses to two practical localized exercise conditions, not as evidence that the plantar-flexion and elbow-flexion tasks produce equivalent systemic biochemical responses when performed at comparable relative intensity or fatigue level.

### 5.8. Study Limitations

Several limitations should be considered when interpreting the present findings. Exercise intensity was not normalized to %MVC, %1RM, power output, or total mechanical work, and movement tempo was not externally paced. In addition, the soleus and biceps brachii differ substantially in muscle mass, architecture, recruitment pattern, functional role, and bilateral versus unilateral activation. Therefore, the observed responses, including the significant cortisol interaction and the nominal glucose interaction, should be interpreted as reflecting two physiologically distinct localized exercise exposures rather than isolated fiber-type effects.

Muscle activation was not measured directly. Consequently, the study cannot quantify the relative contributions of the intended target muscles, synergists, stabilizers, or antagonists during either exercise condition. This does not affect the comparison of the observed systemic biochemical responses between the two applied tasks, but it prevents attribution of those responses to an individual muscle or to muscle-fiber composition. Future mechanistic studies could combine systemic biochemical measurements with normalized multichannel EMG, local blood-flow assessment, or other direct measures of muscle recruitment.

The analyses relied exclusively on circulating biochemical markers, which reflect integrated whole-body regulation rather than direct intramuscular metabolism and may attenuate localized metabolic differences. Although the sample size was relatively large, only the cortisol interaction remained statistically significant after Holm correction. In contrast, the glucose interaction was nominally significant before correction, whereas the remaining biomarkers showed small or non-significant interaction effects after multiple-testing correction.

Another important limitation is the timing of blood sampling. Only one post-exercise blood sample was collected, within 2–3 min after protocol termination. This sampling window captured very early circulating changes but may not have captured peak blood lactate and was insufficient to evaluate delayed CK and LDH kinetics or the full temporal profile of cortisol. Consequently, the study cannot determine whether condition-dependent differences in lactate, enzymatic activity, delayed muscle-damage-related responses, or cortisol would have emerged or changed at later recovery time points. Future studies should include serial blood sampling, for example, immediately post-exercise and at later recovery intervals, to better characterize the time course of enzymatic and hormonal responses.

Hemoglobin and hematocrit were not measured; therefore, exercise-induced changes in plasma volume could not be estimated or used to correct post-exercise biomarker concentrations. Immediate changes in circulating lipid, enzyme, glucose, lactate, and cortisol concentrations may consequently reflect a combination of biological response and transient fluid redistribution. Future studies using similar protocols should include hemoglobin and hematocrit, or another validated method, to estimate exercise-induced plasma-volume change and permit appropriate interpretation or correction of acute circulating biomarker responses.

Sequence allocation followed a predictable alternation rather than concealed randomization; therefore, allocation-related bias cannot be completely excluded. The two sequence groups were numerically balanced (51/51), which reduced imbalance in order assignment but does not by itself exclude carryover.

The revised primary analyses explicitly included study period and assigned sequence, and neither period × time nor sequence × time effects remained significant after multiplicity correction (Supplementary Table S3). Period- and sequence-specific pre-exercise values and within-participant changes between period baselines were also examined (Supplementary Table S5A,B), together with crossover-specific baseline-adjusted and first-period-only sensitivity analyses (Supplementary Table S4). These analyses reduce concern that the primary findings were solely attributable to crossover order; however, they cannot prove the absence of carryover. In particular, the 48–72 h inter-session interval may have been insufficient for complete normalization of delayed CK or LDH activity in some participants. Residual carryover therefore remains possible despite the absence of a consistent period- or sequence-related signal.

## 6. Conclusions

After adjustment for study period and assigned sequence, cortisol retained a statistically significant Holm-corrected condition × time interaction, whereas glucose remained nominally significant but did not meet the multiplicity-corrected significance criterion in the primary model. The period- and sequence-adjusted cortisol estimate was essentially unchanged from the previous unadjusted estimate, suggesting that these design factors did not account for the primary-model interaction. However, crossover-specific, baseline-adjusted, and first-period-only sensitivity analyses provided less consistent support for the cortisol finding, whereas the glucose signal was present in both sensitivity analyses. Accordingly, the cortisol finding should be regarded as a hypothesis-generating early condition-dependent difference in pre-to-post change and not as evidence of a task-specific endocrine profile.

## Figures and Tables

**Table 1:** Characteristics of the study participants (*N* = 102).

Parameter	Value
Sex, *n* male/female	35/67
Age, years	21.31 ± 2.42
Body height, cm	170.87 ± 8.84
Body mass, kg	70.5 ± 17.79
Waist circumference, cm	82.64 ± 12.66
BMI, kg/m^2^	23.95 ± 4.71
WHtR	0.48 ± 0.06
Light physical activity, code 1, *n* (%)	52 (51.0%)
Moderate physical activity, code 2, *n* (%)	19 (18.6%)
High physical activity, code 3, *n* (%)	14 (13.7%)
Very high/intense physical activity, code 4, *n* (%)	17 (16.7%)

Continuous variables are presented as mean ± SD and categorical variables as *n* (%). BMI, body mass index; WHtR, waist-to-height ratio. Physical activity was classified using a study-specific, non-validated four-level ordinal scale: 1, light activity (<2 h/day walking); 2, moderate activity (2–4 h/day walking); 3, high activity (>4 h/day walking); and 4, very high/intense activity, including competitive sport or heavy manual work. This classification was used only for descriptive cohort characterization and should not be interpreted as a validated quantitative measure of habitual physical activity.

**Table 2:** Exercise performance and task-demand characteristics of the plantar-flexion and elbow-flexion tasks.

Variable	Plantar-Flexion Task	Elbow-Flexion Task
Plantar flexion and elbow flexion	Bilateral seated calf raise	Unilateral seated elbow flexion
Intended muscular emphasis	Soleus	Biceps brachii
Limb involvement	Bilateral lower limb	Unilateral dominant upper limb
External load—women	10 kg	2 kg
External load—men	20 kg	4 kg
Repetitions completed—women	42 ± 12	48 ± 14
Repetitions completed—men	65 ± 25	59 ± 10
Exercise duration—men, s	286 ± 28	292 ± 18
Exercise duration—women, s	275 ± 35	286 ± 24
Time per repetition—men, s/rep	4.40	4.95
Time per repetition—women, s/rep	6.55	5.96
Participants reaching objective task failure, *n* (%)	29/102 (28.5%)	22/102 (21.6%)
Participants reaching predefined 5 min cap, *n* (%)	73/102 (71.5%)	80/102 (78.4%)
RPE after exercise—men, Borg CR-10 scale	7.2 ± 1.3	6.4 ± 1.5
RPE after exercise—women, Borg CR-10 scale	6.9 ± 1.4	6.1 ± 1.4
External load–repetition product—men, kg × repetitions	1300 ± 500	236 ± 40
External load–repetition product—women, kg × repetitions	420 ± 120	96 ± 28
Movement tempo	Self-selected; not externally paced	Self-selected; not externally paced
Relative intensity	Not normalized to %MVC or %1RM	Not normalized to %MVC or %1RM
Main interpretive limitation	Larger bilateral lower-limb task; many participants ended by time cap	Smaller unilateral upper-limb task; frequent time-cap completion may indicate weaker effective stimulus

Time per repetition was calculated as exercise duration divided by completed repetitions. The external load–repetition product was calculated as external load × repetitions and is reported only as a descriptive indicator of external loading within each task. It does not incorporate displacement, range of motion, joint mechanics, force throughout the movement, active muscle mass, or bilateral versus unilateral execution; therefore, it should not be interpreted as mechanical work or as a common metric of matched exercise dose between the two tasks.

**Table 3:** Exercise condition × time interaction effects for circulating biochemical outcomes.

Outcome	Interaction Estimate	95% CI	*p*-Value	Holm-Adjusted *p*-Value	Unadjusted Paired Cohen’s d_z_ (95%CI)
TC [mg·dL^−1^]	6.525	−4.930 to 17.980	0.263	1	0.109 (−0.086 to 0.304)
HDL-C [mg·dL^−1^]	−6.303	−18.582 to 5.975	0.313	1	−0.106 (−0.301 to 0.089)
LDL-C [mg·dL^−1^]	−6.901	−17.278 to 3.476	0.192	1	−0.134 (−0.328 to 0.062)
TG [mg·dL^−1^]	−3.75	−17.385 to 9.885	0.589	1	−0.060 (−0.254 to 0.135)
Glucose [mg·dL^−1^]	−7.013	−12.238 to −1.788	0.009	0.078	−0.246 (−0.443 to −0.049)
Lactate [mg·dL^−1^]	0.274	−1.877 to 2.424	0.802	1	0.025 (−0.169 to 0.219)
CK [U·L^−1^]	4.82	−49.754 to 59.394	0.862	1	0.015 (−0.179 to 0.209)
LDH [U·L^−1^]	7.203	−3.815 to 18.221	0.199	1	0.125 (−0.071 to 0.319)
GGT [U·L^−1^]	−0.436	−3.648 to 2.776	0.789	1	−0.025 (−0.219 to 0.169)
Cortisol [nmol·L^−1^]	149.219	62.338 to 236.099	8.21 × 10^−4^	0.008	0.318 (0.118 to 0.516)

Values represent period- and sequence-adjusted model estimates for the exercise condition × time contrast calculated as ΔPlantar − ΔElbow, where Δ denotes the within-condition Post − Pre change. Models included exercise condition, sampling time, study period, assigned sequence, condition × time, period × time, and sequence × time as fixed effects, with participant and participant-specific visit included as random intercepts where supported by the data. Unadjusted and Holm-adjusted *p*-values across the ten predefined primary interaction tests are presented. Cohen’s dz is reported as an unadjusted, paired, standardized difference-of-differences and is provided for descriptive effect-size interpretation; the inferential estimates and *p*-values are derived from the period- and sequence-adjusted mixed-effects models.

## Data Availability

The data supporting the findings of this study are available from the corresponding author upon reasonable request.

## Supplementary Materials

The following supporting information can be downloaded at: https://www.mdpi.com/article/10.3390/life16091525/s1, Table S1: Standardized seven-day meal list and pre-experimental dietary instructions; Table S2: Period- and sequence-adjusted estimated marginal means, condition-specific pre-to-post changes, and pre-exercise between-condition contrasts for circulating biochemical outcomes; Table S3: Period × time and sequence × time effects from the crossover-adjusted mixed-effects models; Table S4: Sensitivity analyses of exercise condition-dependent biochemical changes; Table S5A: Pre-exercise biochemical values according to study period, sequence, and exercise condition; Table S5B: Within-participant period-2 minus period-1 changes in pre-exercise biochemical values, overall and by sequence.
