# Supplementary material for: Immediate Circulating Cortisol Responses Differ Between Two Functionally Divergent Localized Resistance-Type Plantar Flexion and Elbow Flexions: An Exploratory Within-Participant Crossover Study
Source: Life (Basel). 2026 Sep 14;16(9):1525. doi: 10.3390/life16091525 (PMC13608848; doi:10.3390/life16091525)
Supplement: Supplementary file 1 [file life-16-01525-s001.zip › life-4525983-supplementary.pdf]

Supplementary Table S1. Standardized seven-day meal list and pre-experimental dietary instructions

Day	Breakfast	Morning snack	Lunch	Afternoon snack	Evening meal
Day 1	Rolled oats, 5–6 tablespoons, prepared with one glass of milk; one medium banana	One container of natural yogurt and one medium apple	One palm-sized portion of grilled or baked chicken breast; 1–1.5 cups of cooked rice; 1–2 cups of cooked vegetables	Two slices of whole-grain bread with cottage cheese	Two eggs; two slices of whole-grain bread; tomato or cucumber
Day 2	Three slices of whole-grain bread; two boiled eggs; tomato or cucumber	One glass of kefir and one medium banana	One palm-sized portion of roasted turkey; two or three medium boiled potatoes; 1–2 cups of vegetables	One container of natural yogurt with 3–4 tablespoons of rolled oats	Cottage cheese; two or three slices of whole-grain bread; vegetables
Day 3	Five or six tablespoons of unsweetened muesli with one glass of milk or natural yogurt; one apple	One medium banana and one container of natural yogurt	One palm-sized portion of baked white fish; 1–1.5 cups of cooked rice; cooked vegetables	Two slices of whole-grain bread with a mild cheese	Omelette prepared from two eggs; two slices of whole-grain bread; vegetables
Day 4	Rolled oats, 5–6 tablespoons, prepared with milk; one apple or pear	One glass of kefir and one medium banana	One palm-sized portion of chicken or turkey; 1–1.5 cups of cooked buckwheat groats; vegetables	One container of natural yogurt and one piece of fruit	Three slices of whole-grain bread with cottage cheese or lean poultry; vegetables
Day 5	Three slices of whole-grain bread with cottage cheese; one boiled egg; vegetables	One natural yogurt and one apple	One palm-sized portion of baked chicken; 1–1.5 cups of cooked pasta with a mild tomato sauce; vegetables	One banana and one glass of kefir	Two eggs; two or three slices of whole-grain bread; tomato or cucumber
Day 6	Five or six tablespoons of rolled oats with one glass of milk; one banana	One container of natural yogurt and one apple	One palm-sized portion of baked fish or turkey; two or three medium potatoes; vegetables	Two slices of whole-grain bread with cottage cheese	Rice or pasta with a mild tomato sauce and one palm-sized portion of chicken or turkey
Day 7: standardized day preceding each measurement	Rolled oats, 5–6 tablespoons, prepared with one glass of milk; one medium banana	One container of natural yogurt and one medium apple	One palm-sized portion of grilled or baked chicken breast; 1–1.5 cups of cooked white rice; one cup of cooked vegetables	Two slices of whole-grain bread with cottage cheese; water	Two or three slices of whole-grain bread; two boiled eggs; tomato or cucumber. The meal was completed 8–12 h before the scheduled blood collection.

Portions were communicated using household measures because foods were not weighed and total energy and macronutrient intakes were not calculated. The meal list was intended to reduce within-participant dietary variation between experimental sessions rather than to provide identical energy intake across participants. Individual substitutions necessitated by food allergy, intolerance, vegetarian diet, or other dietary restrictions were permitted and documented. The dietary protocol did not permit quantitative evaluation of energy intake, macronutrient intake, or dietary adherence.

Supplementary Table S2. Period- and sequence-adjusted estimated marginal means, condition-specific pre-to-post changes, and pre-exercise between-condition contrasts for circulating biochemical outcomes.

Outcome	Elbow: Pre adjusted EMM (95% CI)	Elbow: Post adjusted EMM (95% CI)	Elbow: Post-Pre adjusted change (95% CI)	Plantar: Pre adjusted EMM (95% CI)	Plantar: Post adjusted EMM (95% CI)	Plantar: Post-Pre adjusted change (95% CI)	Pre Plantar-Elbow contrast (95% CI)	Pre contrast p-value
TC [mg·dL^{-1}]	162.85 (156.90 to 168.80)	161.30 (155.35 to 167.26)	-1.55 (-9.65 to 6.55)	167.09 (161.14 to 173.04)	172.07 (166.12 to 178.02)	4.98 (-3.12 to 13.08)	4.24 (-3.86 to 12.34)	0.304
HDL-C [mg·dL^{-1}]	60.47 (54.33 to 66.60)	66.45 (60.31 to 72.58)	5.98 (-2.70 to 14.66)	62.49 (56.36 to 68.63)	62.17 (56.03 to 68.30)	-0.32 (-9.01 to 8.36)	2.02 (-6.66 to 10.71)	0.647
LDL-C [mg·dL^{-1}]	96.16 (90.84 to 101.48)	95.67 (90.35 to 100.98)	-0.49 (-7.83 to 6.85)	93.66 (88.34 to 98.98)	86.27 (80.95 to 91.58)	-7.39 (-14.73 to -0.06)	-2.50 (-9.84 to 4.84)	0.503
TG [mg·dL^{-1}]	80.54 (73.72 to 87.35)	76.48 (69.67 to 83.30)	-4.05 (-13.69 to 5.59)	82.23 (75.41 to 89.05)	74.43 (67.61 to 81.25)	-7.80 (-17.44 to 1.84)	1.70 (-7.94 to 11.34)	0.73
Glucose [mg·dL^{-1}]	94.73 (92.11 to 97.34)	92.53 (89.92 to 95.14)	-2.20 (-5.89 to 1.50)	93.02 (90.41 to 95.63)	83.81 (81.20 to 86.42)	-9.21 (-12.91 to -5.52)	-1.71 (-5.40 to 1.99)	0.365
Lactate [mg·dL^{-1}]	11.12 (10.02 to 12.23)	12.14 (11.04 to 13.24)	1.02 (-0.50 to 2.54)	8.68 (7.57 to 9.78)	9.97 (8.86 to 11.07)	1.29 (-0.23 to 2.81)	-2.45 (-3.98 to -0.92)	0.002
CK [U·L^{-1}]	127.11 (99.82 to 154.39)	135.11 (107.82 to 162.39)	8.00 (-30.59 to 46.59)	142.88 (115.59 to 170.16)	155.70 (128.41 to 182.98)	12.82 (-25.77 to 51.41)	15.77 (-22.82 to 54.36)	0.422
LDH [U·L^{-1}]	167.22 (161.71 to 172.73)	176.74 (171.23 to 182.25)	9.52 (1.73 to 17.31)	168.62 (163.11 to 174.13)	185.34 (179.84 to 190.85)	16.73 (8.93 to 24.52)	1.40 (-6.39 to 9.19)	0.724
GGT [U·L^{-1}]	15.94 (14.32 to 17.55)	15.86 (14.24 to 17.47)	-0.08 (-2.35 to 2.19)	17.38 (15.77 to 19.00)	16.87 (15.25 to 18.48)	-0.52 (-2.79 to 1.76)	1.45 (-0.82 to 3.72)	0.211
Cortisol [nmol·L^{-1}]	481.96 (437.82 to 526.09)	366.44 (322.30 to 410.58)	-115.51 (-176.95 to -54.08)	424.06 (379.92 to 468.20)	457.76 (413.63 to 501.90)	33.70 (-27.73 to 95.14)	-57.90 (-119.33 to 3.54)	0.065

Values are period- and sequence-adjusted model-estimated marginal means (EMMs), condition-specific Post − Pre contrasts, or pre-exercise Plantar − Elbow contrasts, with 95% confidence intervals. Positive changes indicate an increase and negative changes indicate a decrease. For each biochemical outcome, the model included exercise condition, sampling time, study period, assigned sequence, exercise condition × time, period × time, and sequence × time as fixed effects, with participant and participant-specific study visit included as random intercepts where supported by the model. Supplementary Table S2 and Table 3 were generated from the same period- and sequence-adjusted models and analytical population (N = 102).

Supplementary Table S3. Period × time and sequence × time effects from the crossover-adjusted mixed-effects models.

Outcome	Period x time estimate	Period x time 95% CI	Period x time p-value	Period x time Holm p	Sequence x time estimate	Sequence x time 95% CI	Sequence x time p-value	Sequence x time Holm p
TC [mg·dL^{-1}]	-9.812	-21.267 to 1.643	0.093	0.929	7.727	-3.728 to 19.182	0.185	1
HDL-C [mg·dL^{-1}]	-4.889	-17.167 to 7.390	0.434	1	-4.915	-17.193 to 7.363	0.431	1
LDL-C [mg·dL^{-1}]	-7.781	-18.158 to 2.596	0.141	1	7.44	-2.937 to 17.817	0.159	1
TG [mg·dL^{-1}]	-8.736	-22.371 to 4.899	0.209	1	-9.257	-22.892 to 4.378	0.183	1
Glucose [mg·dL^{-1}]	-0.806	-6.031 to 4.420	0.762	1	2.024	-3.201 to 7.250	0.447	1
Lactate [mg·dL^{-1}]	-1.228	-3.378 to 0.923	0.262	1	-1.293	-3.444 to 0.857	0.237	1
CK [U·L^{-1}]	-36.639	-91.212 to 17.935	0.188	1	-28.332	-82.906 to 26.242	0.308	1
LDH [U·L^{-1}]	-6.817	-17.835 to 4.201	0.225	1	-5.938	-16.956 to 5.080	0.29	1
GGT [U·L^{-1}]	-2.504	-5.715 to 0.708	0.126	1	1.313	-1.899 to 4.525	0.422	1
Cortisol [nmol·L^{-1}]	-22.733	-109.613 to 64.148	0.607	1	-81.454	-168.334 to 5.427	0.066	0.66

Supplementary Table S4. Sensitivity analyses of exercise condition-dependent biochemical changes.

Outcome	Analysis	Estimand	Estimate	95% CI	p-value	Holm-adjusted p-value within analysis family
TC [mg·dL-1]	Crossover baseline-adjusted ANCOVA sensitivity	Post Plantar-Elbow difference at Pre Plantar-Elbow difference = 0, averaged over sequence	10.826	2.504 to 19.148	0.011	0.09
TC [mg·dL-1]	First-period-only sensitivity	Period-1 Plantar minus Elbow pre-to-post change	14.252	-2.656 to 31.159	0.098	0.878
TC [mg·dL-1]	Previous unadjusted LMM	(Plantar Post-Pre) - (Elbow Post-Pre)	6.525	-5.082 to 18.132	0.269	1
TC [mg·dL-1]	Primary period- and sequence-adjusted LMM	(Plantar Post-Pre) - (Elbow Post-Pre)	6.525	-4.930 to 17.980	0.263	1
HDL-C [mg·dL-1]	Crossover baseline-adjusted ANCOVA sensitivity	Post Plantar-Elbow difference at Pre Plantar-Elbow difference = 0, averaged over sequence	-5.836	-17.615 to 5.943	0.328	1
HDL-C [mg·dL-1]	First-period-only sensitivity	Period-1 Plantar minus Elbow pre-to-post change	-11.219	-34.111 to 11.674	0.333	1
HDL-C [mg·dL-1]	Previous unadjusted LMM	(Plantar Post-Pre) - (Elbow Post-Pre)	-6.303	-18.560 to 5.953	0.313	1
HDL-C [mg·dL-1]	Primary period- and sequence-adjusted LMM	(Plantar Post-Pre) - (Elbow Post-Pre)	-6.303	-18.582 to 5.975	0.313	1
LDL-C [mg·dL-1]	Crossover baseline-adjusted ANCOVA sensitivity	Post Plantar-Elbow difference at Pre Plantar-Elbow difference = 0, averaged over sequence	-9.335	-16.553 to -2.118	0.012	0.09
LDL-C [mg·dL-1]	First-period-only sensitivity	Period-1 Plantar minus Elbow pre-to-post change	0.539	-14.331 to 15.408	0.943	1
LDL-C [mg·dL-1]	Previous unadjusted LMM	(Plantar Post-Pre) - (Elbow Post-Pre)	-6.901	-17.402 to 3.600	0.197	1
LDL-C [mg·dL-1]	Primary period- and sequence-adjusted LMM	(Plantar Post-Pre) - (Elbow Post-Pre)	-6.901	-17.278 to 3.476	0.192	1
TG [mg·dL-1]	Crossover baseline-adjusted ANCOVA sensitivity	Post Plantar-Elbow difference at Pre Plantar-Elbow difference = 0, averaged over sequence	-2.059	-10.236 to 6.117	0.618	1
TG [mg·dL-1]	First-period-only sensitivity	Period-1 Plantar minus Elbow pre-to-post change	-13.007	-33.231 to 7.218	0.205	1
TG [mg·dL-1]	Previous unadjusted LMM	(Plantar Post-Pre) - (Elbow Post-Pre)	-3.75	-17.381 to 9.881	0.589	1
TG [mg·dL-1]	Primary period- and sequence-adjusted LMM	(Plantar Post-Pre) - (Elbow Post-Pre)	-3.75	-17.385 to 9.885	0.589	1
Glucose [mg·dL-1]	Crossover baseline-adjusted ANCOVA sensitivity	Post Plantar-Elbow difference at Pre Plantar-Elbow difference = 0, averaged over sequence	-8.918	-12.406 to -5.430	1.83E-06	1.83E-05
Glucose [mg·dL-1]	First-period-only sensitivity	Period-1 Plantar minus Elbow pre-to-post change	-4.989	-8.306 to -1.671	0.004	0.036
Glucose [mg·dL-1]	Previous unadjusted LMM	(Plantar Post-Pre) - (Elbow Post-Pre)	-7.013	-12.233 to -1.793	0.009	0.078

Glucose [mg·dL-1]	Primary period- and sequence-adjusted LMM	(Plantar Post-Pre) - (Elbow Post-Pre)	-7.013	-12.238 to -1.788	0.009	0.078
Lactate [mg·dL-1]	Crossover baseline-adjusted ANCOVA sensitivity	Post Plantar-Elbow difference at Pre Plantar-Elbow difference = 0, averaged over sequence	-2.075	-3.878 to -0.272	0.025	0.142
Lactate [mg·dL-1]	First-period-only sensitivity	Period-1 Plantar minus Elbow pre-to-post change	-1.02	-3.779 to 1.740	0.465	1
Lactate [mg·dL-1]	Previous unadjusted LMM	(Plantar Post-Pre) - (Elbow Post-Pre)	0.274	-1.889 to 2.437	0.804	1
Lactate [mg·dL-1]	Primary period- and sequence-adjusted LMM	(Plantar Post-Pre) - (Elbow Post-Pre)	0.274	-1.877 to 2.424	0.802	1
CK [U·L-1]	Crossover baseline-adjusted ANCOVA sensitivity	Post Plantar-Elbow difference at Pre Plantar-Elbow difference = 0, averaged over sequence	22.906	-19.363 to 65.174	0.285	1
CK [U·L-1]	First-period-only sensitivity	Period-1 Plantar minus Elbow pre-to-post change	-23.512	-102.342 to 55.318	0.555	1
CK [U·L-1]	Previous unadjusted LMM	(Plantar Post-Pre) - (Elbow Post-Pre)	4.82	-49.670 to 59.310	0.862	1
CK [U·L-1]	Primary period- and sequence-adjusted LMM	(Plantar Post-Pre) - (Elbow Post-Pre)	4.82	-49.754 to 59.394	0.862	1
LDH [U·L-1]	Crossover baseline-adjusted ANCOVA sensitivity	Post Plantar-Elbow difference at Pre Plantar-Elbow difference = 0, averaged over sequence	8.677	1.185 to 16.170	0.024	0.142
LDH [U·L-1]	First-period-only sensitivity	Period-1 Plantar minus Elbow pre-to-post change	1.265	-15.892 to 18.422	0.884	1
LDH [U·L-1]	Previous unadjusted LMM	(Plantar Post-Pre) - (Elbow Post-Pre)	7.203	-3.805 to 18.211	0.199	1
LDH [U·L-1]	Primary period- and sequence-adjusted LMM	(Plantar Post-Pre) - (Elbow Post-Pre)	7.203	-3.815 to 18.221	0.199	1
GGT [U·L-1]	Crossover baseline-adjusted ANCOVA sensitivity	Post Plantar-Elbow difference at Pre Plantar-Elbow difference = 0, averaged over sequence	1.167	-1.184 to 3.519	0.327	1
GGT [U·L-1]	First-period-only sensitivity	Period-1 Plantar minus Elbow pre-to-post change	0.876	-3.257 to 5.010	0.675	1
GGT [U·L-1]	Previous unadjusted LMM	(Plantar Post-Pre) - (Elbow Post-Pre)	-0.436	-3.667 to 2.795	0.791	1
GGT [U·L-1]	Primary period- and sequence-adjusted LMM	(Plantar Post-Pre) - (Elbow Post-Pre)	-0.436	-3.648 to 2.776	0.789	1
Cortisol [nmol·L-1]	Crossover baseline-adjusted ANCOVA sensitivity	Post Plantar-Elbow difference at Pre Plantar-Elbow difference = 0, averaged over sequence	87.784	25.690 to 149.879	0.006	0.054
Cortisol [nmol·L-1]	First-period-only sensitivity	Period-1 Plantar minus Elbow pre-to-post change	67.765	-59.692 to 195.222	0.294	1
Cortisol [nmol·L-1]	Previous unadjusted LMM	(Plantar Post-Pre) - (Elbow Post-Pre)	149.219	62.238 to 236.199	8.32E-04	0.008
Cortisol [nmol·L-1]	Primary period- and sequence-adjusted LMM	(Plantar Post-Pre) - (Elbow Post-Pre)	149.219	62.338 to 236.099	8.21E-04	0.008

Supplementary Table S5A. Pre-exercise biochemical values according to study period, sequence, and exercise condition.

Outcome	Period	Sequence	Condition	n	Mean (SD)	Median (Q1-Q3)	Range
TC [mg·dL^{-1}]	Period 1	Elbow_then_Plantar	Elbow flexion	51	166.21 (34.69)	160.00 (143.00-189.50)	113.00 to 300.00
TC [mg·dL^{-1}]	Period 1	Plantar_then_Elbow	Plantar flexion	51	166.26 (31.27)	162.00 (144.50-188.50)	97.90 to 238.00
TC [mg·dL^{-1}]	Period 2	Elbow_then_Plantar	Plantar flexion	51	167.92 (27.60)	164.00 (148.50-183.50)	119.00 to 250.00
TC [mg·dL^{-1}]	Period 2	Plantar_then_Elbow	Elbow flexion	51	159.49 (28.16)	157.00 (139.00-175.50)	112.00 to 241.00
HDL-C [mg·dL^{-1}]	Period 1	Elbow_then_Plantar	Elbow flexion	51	60.97 (9.89)	60.20 (53.95-69.55)	35.90 to 79.90
HDL-C [mg·dL^{-1}]	Period 1	Plantar_then_Elbow	Plantar flexion	51	63.29 (13.69)	61.60 (54.80-73.35)	34.60 to 96.50
HDL-C [mg·dL^{-1}]	Period 2	Elbow_then_Plantar	Plantar flexion	51	61.70 (13.57)	59.50 (52.75-70.65)	32.20 to 95.60
HDL-C [mg·dL^{-1}]	Period 2	Plantar_then_Elbow	Elbow flexion	51	59.96 (13.48)	57.90 (49.95-70.30)	28.90 to 95.50
LDL-C [mg·dL^{-1}]	Period 1	Elbow_then_Plantar	Elbow flexion	51	99.75 (31.27)	93.20 (79.75-115.00)	60.10 to 224.00
LDL-C [mg·dL^{-1}]	Period 1	Plantar_then_Elbow	Plantar flexion	51	92.70 (27.35)	87.00 (73.50-111.31)	41.90 to 160.00
LDL-C [mg·dL^{-1}]	Period 2	Elbow_then_Plantar	Plantar flexion	51	94.62 (27.56)	89.80 (76.25-111.00)	41.90 to 174.00
LDL-C [mg·dL^{-1}]	Period 2	Plantar_then_Elbow	Elbow flexion	51	92.57 (25.30)	87.60 (74.00-107.50)	51.60 to 161.00
TG [mg·dL^{-1}]	Period 1	Elbow_then_Plantar	Elbow flexion	51	76.07 (28.96)	65.60 (52.80-100.90)	39.00 to 132.00
TG [mg·dL^{-1}]	Period 1	Plantar_then_Elbow	Plantar flexion	51	83.93 (37.25)	73.40 (54.95-101.50)	34.10 to 184.00
TG [mg·dL^{-1}]	Period 2	Elbow_then_Plantar	Plantar flexion	51	80.53 (30.83)	74.30 (57.55-98.45)	41.30 to 162.00
TG [mg·dL^{-1}]	Period 2	Plantar_then_Elbow	Elbow flexion	51	85.00 (46.37)	72.70 (54.00-94.95)	34.40 to 293.00
Glucose [mg·dL^{-1}]	Period 1	Elbow_then_Plantar	Elbow flexion	51	93.75 (6.57)	92.00 (89.00-98.50)	83.00 to 110.00
Glucose [mg·dL^{-1}]	Period 1	Plantar_then_Elbow	Plantar flexion	51	92.32 (6.38)	91.00 (88.00-96.50)	78.00 to 109.00
Glucose [mg·dL^{-1}]	Period 2	Elbow_then_Plantar	Plantar flexion	51	93.73 (5.86)	93.00 (89.50-97.00)	83.00 to 108.00
Glucose [mg·dL^{-1}]	Period 2	Plantar_then_Elbow	Elbow flexion	51	95.71 (25.45)	91.00 (88.50-95.50)	81.00 to 269.00
Lactate [mg·dL^{-1}]	Period 1	Elbow_then_Plantar	Elbow flexion	51	9.63 (3.66)	8.84 (7.44-11.10)	4.92 to 22.20
Lactate [mg·dL^{-1}]	Period 1	Plantar_then_Elbow	Plantar flexion	51	8.76 (3.40)	8.20 (7.11-9.49)	4.17 to 27.20
Lactate [mg·dL^{-1}]	Period 2	Elbow_then_Plantar	Plantar flexion	51	8.59 (3.05)	7.91 (6.86-9.25)	5.38 to 22.20
Lactate [mg·dL^{-1}]	Period 2	Plantar_then_Elbow	Elbow flexion	51	12.62 (7.56)	11.30 (8.53-14.65)	3.59 to 51.50
CK [U·L^{-1}]	Period 1	Elbow_then_Plantar	Elbow flexion	51	110.75 (85.77)	81.30 (65.35-104.00)	32.10 to 409.00
CK [U·L^{-1}]	Period 1	Plantar_then_Elbow	Plantar flexion	51	140.72 (160.77)	89.60 (63.00-134.50)	37.60 to 905.00
CK [U·L^{-1}]	Period 2	Elbow_then_Plantar	Plantar flexion	51	145.03 (137.95)	95.70 (68.95-133.50)	32.50 to 573.00
CK [U·L^{-1}]	Period 2	Plantar_then_Elbow	Elbow flexion	51	143.46 (141.76)	91.30 (60.80-149.50)	40.00 to 777.00
LDH [U·L^{-1}]	Period 1	Elbow_then_Plantar	Elbow flexion	51	162.41 (37.20)	160.00 (146.00-182.47)	15.50 to 300.00
LDH [U·L^{-1}]	Period 1	Plantar_then_Elbow	Plantar flexion	51	168.77 (25.19)	166.00 (154.50-179.50)	130.00 to 262.00
LDH [U·L^{-1}]	Period 2	Elbow_then_Plantar	Plantar flexion	51	168.47 (22.59)	170.00 (155.50-182.50)	127.00 to 224.00
LDH [U·L^{-1}]	Period 2	Plantar_then_Elbow	Elbow flexion	51	172.02 (24.11)	166.00 (157.50-184.50)	111.00 to 229.00
GGT [U·L^{-1}]	Period 1	Elbow_then_Plantar	Elbow flexion	51	14.78 (6.26)	14.00 (10.85-16.00)	7.20 to 37.80
GGT [U·L^{-1}]	Period 1	Plantar_then_Elbow	Plantar flexion	51	15.73 (7.56)	14.20 (10.50-17.80)	5.40 to 40.10
GGT [U·L^{-1}]	Period 2	Elbow_then_Plantar	Plantar flexion	51	19.03 (11.74)	15.40 (11.70-22.60)	6.80 to 70.40
GGT [U·L^{-1}]	Period 2	Plantar_then_Elbow	Elbow flexion	51	17.09 (6.33)	16.20 (11.75-22.10)	7.10 to 32.50
Cortisol [nmol·L^{-1}]	Period 1	Elbow_then_Plantar	Elbow flexion	51	468.42 (232.92)	403.00 (319.50-543.50)	142.00 to 1169.00
Cortisol [nmol·L^{-1}]	Period 1	Plantar_then_Elbow	Plantar flexion	51	431.20 (236.79)	354.00 (282.00-478.00)	170.00 to 1288.00
Cortisol [nmol·L^{-1}]	Period 2	Elbow_then_Plantar	Plantar flexion	51	416.92 (187.36)	364.00 (290.50-503.50)	170.00 to 957.00
Cortisol [nmol·L^{-1}]	Period 2	Plantar_then_Elbow	Elbow flexion	51	495.49 (262.10)	453.00 (325.50-581.50)	131.00 to 1750.00

Supplementary Table S5B. Within-participant period-2 minus period-1 changes in pre-exercise biochemical values, overall and by sequence.

Outcome	Stratum	n	Mean Period2-Period1 baseline shift (SD)	Median shift (Q1-Q3)
TC [mg·dL^{-1}]	All participants	102	-2.53 (41.30)	-0.50 (-25.75-22.75)
TC [mg·dL^{-1}]	Elbow_then_Plantar	51	1.71 (43.70)	7.00 (-23.50-28.50)
TC [mg·dL^{-1}]	Plantar_then_Elbow	51	-6.77 (38.72)	-2.00 (-36.00-16.00)
TC [mg·dL^{-1}]	All participants	102	-1.30 (17.56)	-1.10 (-15.18-11.57)
HDL-C [mg·dL^{-1}]	Elbow_then_Plantar	51	0.72 (16.68)	0.50 (-13.60-13.10)
HDL-C [mg·dL^{-1}]	Plantar_then_Elbow	51	-3.33 (18.33)	-1.80 (-16.20-9.25)
HDL-C [mg·dL^{-1}]	All participants	102	-2.63 (36.97)	-0.60 (-25.15-17.67)
HDL-C [mg·dL^{-1}]	Elbow_then_Plantar	51	-5.13 (40.92)	-1.00 (-25.70-16.75)
LDL-C [mg·dL^{-1}]	Plantar_then_Elbow	51	-0.13 (32.76)	0.38 (-19.25-17.95)
LDL-C [mg·dL^{-1}]	All participants	102	2.76 (47.10)	1.51 (-27.05-29.25)
LDL-C [mg·dL^{-1}]	Elbow_then_Plantar	51	4.46 (43.43)	3.80 (-29.85-28.50)
LDL-C [mg·dL^{-1}]	Plantar_then_Elbow	51	1.06 (50.88)	-4.40 (-24.90-28.55)
TG [mg·dL^{-1}]	All participants	102	1.68 (20.14)	0.00 (-6.00-5.00)
TG [mg·dL^{-1}]	Elbow_then_Plantar	51	-0.02 (9.25)	1.00 (-6.00-4.50)
TG [mg·dL^{-1}]	Plantar_then_Elbow	51	3.39 (26.97)	-1.00 (-6.00-5.50)
TG [mg·dL^{-1}]	All participants	102	1.41 (7.27)	0.64 (-2.32-3.15)
Glucose [mg·dL^{-1}]	Elbow_then_Plantar	51	-1.04 (4.27)	-0.70 (-3.08-1.55)
Glucose [mg·dL^{-1}]	Plantar_then_Elbow	51	3.86 (8.74)	2.75 (-0.66-6.97)
Glucose [mg·dL^{-1}]	All participants	102	18.51 (197.59)	7.15 (-32.05-64.65)
Glucose [mg·dL^{-1}]	Elbow_then_Plantar	51	34.28 (163.70)	4.00 (-31.90-67.75)
Lactate [mg·dL^{-1}]	Plantar_then_Elbow	51	2.75 (227.07)	9.00 (-39.45-48.90)
Lactate [mg·dL^{-1}]	All participants	102	4.66 (41.19)	4.00 (-16.50-25.50)
Lactate [mg·dL^{-1}]	Elbow_then_Plantar	51	6.06 (45.85)	4.00 (-17.50-22.50)
Lactate [mg·dL^{-1}]	Plantar_then_Elbow	51	3.26 (36.34)	2.05 (-14.00-26.00)
CK [U·L^{-1}]	All participants	102	2.81 (11.67)	2.40 (-1.05-7.35)
CK [U·L^{-1}]	Elbow_then_Plantar	51	4.25 (13.81)	2.60 (-1.15-9.55)
CK [U·L^{-1}]	Plantar_then_Elbow	51	1.36 (8.95)	2.40 (-1.00-6.10)
CK [U·L^{-1}]	All participants	102	6.40 (338.01)	-1.00 (-122.75-140.50)
LDH [U·L^{-1}]	Elbow_then_Plantar	51	-51.50 (288.96)	-15.00 (-156.00-74.00)
LDH [U·L^{-1}]	Plantar_then_Elbow	51	64.29 (374.76)	45.00 (-74.50-227.50)
